# TAOK2 rescues autism-linked developmental deficits in a 16p11.2 microdeletion mouse model

**DOI:** 10.1038/s41380-022-01785-3

**Published:** 2022-09-19

**Authors:** Robin Scharrenberg, Melanie Richter, Ole Johanns, Durga Praveen Meka, Tabitha Rücker, Nadeem Murtaza, Zsuzsa Lindenmaier, Jacob Ellegood, Anne Naumann, Bing Zhao, Birgit Schwanke, Jan Sedlacik, Jens Fiehler, Ileana L. Hanganu-Opatz, Jason P. Lerch, Karun K. Singh, Froylan Calderon de Anda

**Affiliations:** 1 Institute of Developmental Neurophysiology, Center for Molecular Neurobiology, University Medical Center Hamburg-Eppendorf, 20251 Hamburg, Germany; 2 Krembil Research Institute, Donald K. Johnson Eye Institute, University Health Network, 60 Leonard Ave, Toronto, ON M5T 0S8 Canada; 3 Faculty of Medicine, University of Toronto, Medical Sciences Building, 1 King’s College Cir, Toronto, ON M5S 1A8 Canada; 4 Department of Biochemistry and Biomedical Sciences, Faculty of Health Sciences, McMaster University, Hamilton, ON L8S 4A9 Canada; 5 Mouse Imaging Centre, Hospital for Sick Children, Toronto, ON M5T 3H7 Canada; 6 Department of Medical Biophysics, University of Toronto, Toronto, ON M5S 1A1 Canada; 7 Department of Neuroradiology, University Medical Center Hamburg-Eppendorf (UKE), Hamburg, Germany; 8 Wellcome Centre for Integrative Neuroimaging, The University of Oxford, Oxford, OX3 9DU UK

**Keywords:** Neuroscience, Autism spectrum disorders

## Abstract

The precise development of the neocortex is a prerequisite for higher cognitive and associative functions. Despite numerous advances that have been made in understanding neuronal differentiation and cortex development, our knowledge regarding the impact of specific genes associated with neurodevelopmental disorders on these processes is still limited. Here, we show that Taok2, which is encoded in humans within the autism spectrum disorder (ASD) susceptibility locus 16p11.2, is essential for neuronal migration. Overexpression of de novo mutations or rare variants from ASD patients disrupts neuronal migration in an isoform-specific manner. The mutated TAOK2α variants but not the TAOK2β variants impaired neuronal migration. Moreover, the TAOK2α isoform colocalizes with microtubules. Consequently, neurons lacking Taok2 have unstable microtubules with reduced levels of acetylated tubulin and phosphorylated JNK1. Mice lacking Taok2 develop gross cortical and cortex layering abnormalities. Moreover, acute Taok2 downregulation or Taok2 knockout delayed the migration of upper-layer cortical neurons in mice, and the expression of a constitutively active form of JNK1 rescued these neuronal migration defects. Finally, we report that the brains of the Taok2 KO and 16p11.2 del Het mouse models show striking anatomical similarities and that the heterozygous 16p11.2 microdeletion mouse model displayed reduced levels of phosphorylated JNK1 and neuronal migration deficits, which were ameliorated upon the introduction of TAOK2α in cortical neurons and in the developing cortex of those mice. These results delineate the critical role of TAOK2 in cortical development and its contribution to neurodevelopmental disorders, including ASD.

## Introduction

Extensive population-based screening efforts in disease genetics aim to shed light on the complex forms of neurodevelopmental disorders, e.g., autism spectrum disorder (ASD) and schizophrenia. Susceptibility loci with copy number variants (CNVs), such as 16p11.2, have been identified in cohorts of autistic patients. Recurrent microdeletion and a reciprocal microduplication of this chromosomal region confer increased susceptibility to ASD and schizophrenia, respectively, and contribute to ~1% of all cases of ASD [1]. In particular, the 16p11.2 protein interaction network and spatiotemporal gene expression analysis revealed that the late mid-fetal period is critical for establishing connectivity of 16p11.2 proteins in humans, thus suggesting a role in controlling brain size [2]. Importantly, the cortex volume of 16p11.2 CNV carriers are altered [3]; specifically, the 16p11.2 microdeletion reduces cortex thickness.

One of the ~30 genes encoded in the genomic 16p11.2 stretch is the serine/threonine protein kinase Thousand and one amino-acid kinase 2 (TAOK2). Alternative splicing of TAOK2 results in two isoforms that share exons 1–16 [4]; these isoforms, TAOK2α and TAOK2β, presumably have distinct functional roles. Previously, we showed that defective Neuropilin 1 (Nrp1)-TAOK2-JNK1 signals reduced the production of basal dendrites in the cortex [5]. Furthermore, Taok2 controls hippocampal spine morphology via p38 and Arcadlin/N-Cadherin [4] as well as dendrite recruitment of Myosin Va acting downstream of MST3–Tao1/2 activation [6] and spine maturation [7]. Thus, TAOK2 has been implicated in neurodevelopmental disorders and is listed as a category 2-risk gene (strong association) of the SFARI GENE Scoring list (https://gene.sfari.org/database/human-gene/TAOK2). Moreover, whole-genome and exome sequencing of ASD families identified 24 different variants in TAOK2, of which 3 are de novo mutations [8].

Recently, we found gene-dosage-dependent impairments in cognition, anxiety, and social interaction in Taok2 heterozygous (Het) and knockout (KO) mice, highlighting that a loss of Taok2 produces ASD-like phenotypes. Taok2 Het and KO mice also have dosage-dependent abnormalities in brain size in multiple regions and deficits in upper cortical layering [8], demonstrating the potential importance of this 16p11.2-encoded gene for late cortical development. Specifically, we reported that Taok2 mutant mice have reduced cortex thickness, which correlates with decreased thickness of upper-layer neurons positive for the transcription factor Cux-1. In addition, Cux-1-positive cells are clustered in the more superficial upper cortex, especially in the medial dorsal cortex. Given that the density of Cux-1-positive cells is not affected in KO cortices [8], it is conceivable that neuronal migration defects might account for these cytoarchitectural abnormalities.

Moreover, the expression of TAOK2 was not associated with neurogenesis in human fetal cortices [9]. Other than TAOK2, as shown in this study, no single gene from the 16p11.2 chromosomal region has been linked to brain morphological abnormalities thus far [10]. We show that loss of TAOK2α disturbs neuronal migration presumably by affecting microtubule acetylation and microtubule dynamics through JNK1 phosphorylation in the murine Het 16p11.2 microdeletion model for ASD. Ectopic expression of wild-type (WT) TAOK2α, but not an ASD-associated human variant of TAOK2α, can rescue the migration deficit in the Het 16p11.2 background, making TAOK2 a significant contributor to the anomalies in brain size observed in 16p11.2 CNV carriers [3].

## Results

### ASD-associated TAOK2 mutations affect neuronal positioning during cortex development in an isoform-specific manner

Neuronal migration defects have been associated with multiple disorders related to development, such as ASD [11]. Therefore, we asked whether TAOK2 de novo missense mutations and rare inherited variants recently identified by us in ASD subjects [8] affect neuronal migration. We tested two de novo mutations and one rare inherited variant. Of the two de novo mutations, one is exclusively in TAOK2β (P1022*; C-terminal frameshift deletion resulting in truncation). The other de novo mutation (A135P) and the inherited rare variant (A335V) are present in both isoforms (TAOK2α and TAOK2β) and are localized within the kinase domain or close to it, respectively. The A135P mutation renders TAOK2 a kinase-inactive protein [8]. Notably, phospho-Taok2 levels increase during upper-layer neuronal migration (E15-P1) and decrease in the early postnatal days (Supplementary Fig. 1), implying that Taok2 kinase activity is likely relevant during the constitution of upper cortical layers. Consequently, we did not detect any differences in the distribution of deep layer neurons in mice lacking Taok2 (Supplementary Fig. 2).

**Fig. 1:**
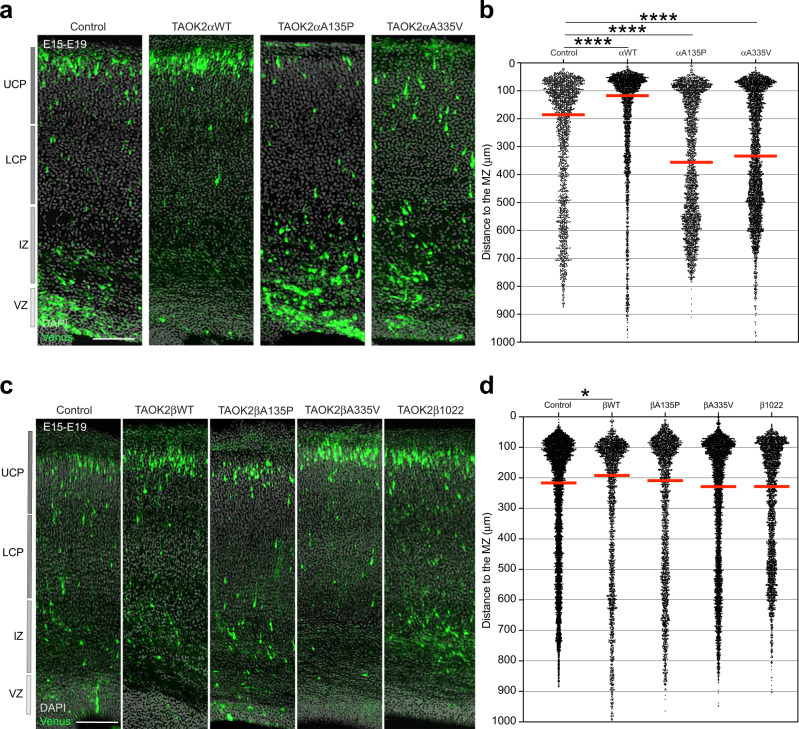
TAOK2α, but not TAOK2β, controls neuronal migration in the developing cortex. **a**, **c** Expression of ASD-associated mutations in TAOK2α, but not in TAOK2β, affects neuronal distribution in the cortex, with cells bearing functional mutations in TAOK2α arrested in the IZ. **b** Quantification of cell distribution in the developing cortex shows cells accumulating in the IZ after TAOK2αA135P and TAOK2αA335V expression compared to control-transfected cortices or cells expressing TAOK2αWT (*p* < 0.0001 by one-way ANOVA, post hoc Dunnett’s multiple test ****p < 0.0001; control = 3 brains, TAOK2α WT = 3 brains, TAOK2αA135P = 4 brains, TAOK2αA335V = 4 brains; median is represented by red line; see Supplementary Fig. 3a for individual brains). **d** Quantification of cell distribution in the developing cortex shows unaffected neuronal migration after TAOK2βA135P, TAOK2βA335V, and TAOK2β1022 expression compared to control-transfected cortices or cells expressing TAOK2βWT (*p* = 0.0002 by one-way ANOVA, post hoc Dunnett’s multiple test *p = 0.0138; control = 5 brains, TAOK2βWT = 4 brains, TAOK2βA135P = 4 brains, TAOK2βA335V = 4 brains, TAOK2βA1022 = 4 brains; median is represented by red line; see Supplementary Fig. 3b for individual brains). Scale bar: 200 μm.

Therefore, we introduced TAOK2 mutations together with a Venus-expressing plasmid into cortical progenitor cells by in utero electroporation at E15 to analyze upper-layer neurons. At E19, brains were harvested, fixed, and subjected to immunostaining. Interestingly, only TAOK2α mutations and not TAOK2β mutations resulted in altered cell positioning within the cortices of E19 mice with fewer transfected cells within the cortical plate (CP). In contrast, the rate of transfected cells in the intermediate zone (IZ) increased compared with that of control cells (Fig. 1a–d and Supplementary Fig. 3 for the single brains analyzed). These results suggest that TAOK2 modulates neuronal migration in an isoform-specific manner.

To gain more insight into differences between TAOK2α and TAOK2β that might account for the neuronal mispositioning defects we detected during neuronal migration, we expressed the α- and β-isoforms in dissociated cortical neurons and analyzed their subcellular distribution using a TAOK2-specific antibody. Analysis of cultured cortical at day in vitro (DIV) 14 revealed that TAOK2α colocalizes with acetylated tubulin but not with actin-rich dendritic protrusions, as indicated by co-staining with rhodamine-labeled phalloidin (Supplementary Fig. 4a, c). On the other hand, TAOK2β is not localized to acetylated microtubules but adjacent to them (Supplementary Fig. 4b, c) and, as previously shown, is present in dendritic filopodia (Supplementary Fig. 4b and [8]). Moreover, we found that lack of Taok2 affected the distribution of endoplasmic reticulum in migrating neurons in the developing cortex (Supplementary Fig. 4d, e), suggesting an impact of Taok2 on ER integrity and microtubule dynamics given that TAOK2α tethers microtubules to the endoplasmic reticulum and that lack of TAOK2 significantly increases microtubule dynamics in a TAOK2 KO HEK293T cell line [12]. Taken together, our results suggest that TAOK2 might modulate microtubule dynamics in an isoform-specific manner through TAOK2α. Accordingly, we found that the expression of TAOK2αA135P, but not TAOK2βA135P, in neuroblastoma cells (SHSY5Y) increased the motility speed of EB3 comets (labeled with mCherry-tagged EB3 plasmid) compared with cells expressing TAOK2α WT or EB3-mCherry alone (Supplementary Fig. 5). In this regard, it was shown that Taxol, which stabilizes microtubules, reduces EB3 comet speed in growing axons [13]. Moreover, growing neurites exhibit decreased EB3 speed, which correlates with stable microtubules containing increased acetylated tubulin levels [14–16]. Thus, our results show that a lack of TAOK2 activity affects microtubule dynamics.

**Fig. 2:**
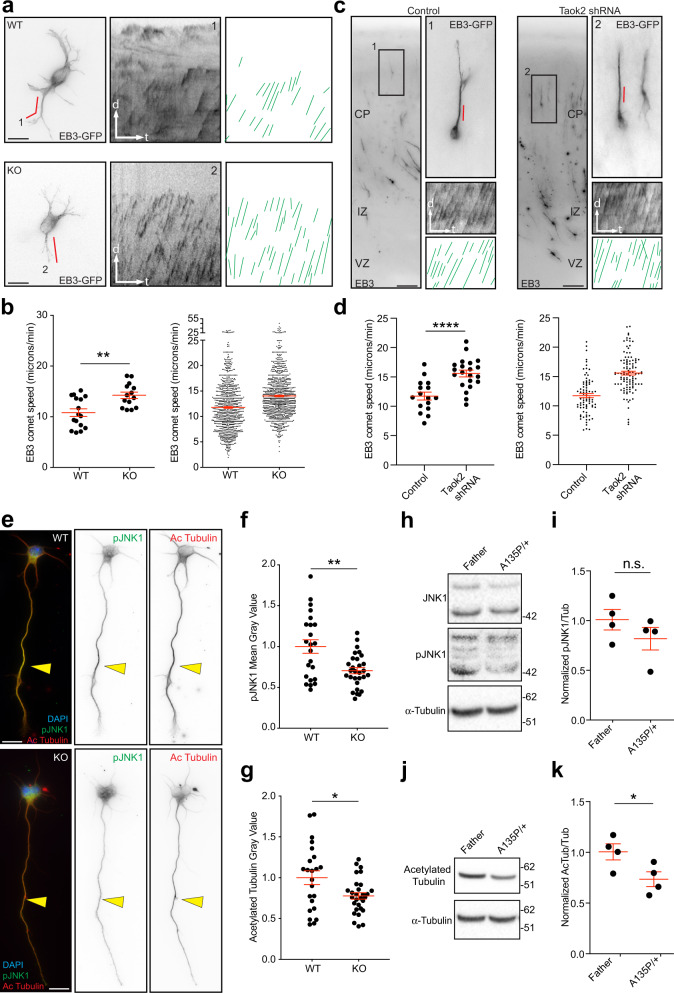
Taok2 modulates microtubule stability. Kymographs of EB3-GFP signals along neurites from WT (upper) and Taok2 KO cells (lower panel) in dissociated cultures in **a** or from control shRNA (left) and Taok2 shRNA-transfected cells (right panel) in acute slices in **c**. Kymograph analysis shows an overall change in the trajectories of EB3 comets (green lines) with steeper trajectories in Taok2 KO or Taok2 shRNA-transfected cells. Left graph: quantification of EB3 speed in the neurite shaft in dissociated cells (**b**) or the leading process of migrating neurons (**d**). Taok2 KO cells and Taok2 shRNA-transfected cells show an increased EB3 speed. Right graph: all values for EB3 speed from the individual cells. (**b** = **p < 0.001 by unpaired *t*-test; *n* = 16 (WT) and 14 (KO) cells from three different brains; values are mean ± s.e.m; **d** = ****p < 0.0001 by unpaired *t-*test; *n* = 16 (control) and 21 (Taok2 shRNA) cells from three different brains; values are mean ± s.e.m.). **e** Cultured cortical neurons (2 DIV) from WT (upper) and Taok2 KO (lower panel) brains show less pJNK1 (green) and less acetylated tubulin (red) expression in Taok2 KO cells compared with WT cells (yellow arrowheads). **f** Quantification of pJNK1 content in the longest neurite. Taok2 KO cells have reduced pJNK1 expression levels (**p = 0.0013 by unpaired *t-*test; WT = 15 cells and KO = 17 cells from three cultures; values are mean ± s.e.m.). **g** Quantification of acetylated tubulin content in the longest neurite. Taok2 KO cells have reduced acetylated tubulin expression levels (*p = 0.0163 by unpaired *t-*test; WT = 15 cells and KO = 17 cells from three cultures; values are mean ± s.e.m.). Western blot analysis (**h**) and quantification (**i**) of pJNK1 from LCLs of the TAOK2αA135P Proband and the Father; *p* = 0.259 by *t-*test; *n* = 4. Western blot analysis (**j**) and quantification (**k**) of acetylated tubulin from LCLs of the TAOK2αA135P Proband and the Father; *p = 0.046 by unpaired *t-*test; *n* = 4. Scale bar: 10 μm.

To further test this hypothesis, we analyzed microtubule dynamics by time-lapse imaging in cultured Taok2-deficient (KO) cortical neurons and Taok2-deficient migrating neurons (after acute knockdown with a Taok2 shRNA) in the developing cortex. To this end, dissociated cells from KO or WT cortices were transfected with EB3-GFP plasmid before plating to label plus-end microtubules. To track growing microtubules in situ, we transfected cortical neurons in utero with Taok2 shRNA or control shRNA together with EB3-GFP at E15. Cortical slices were prepared for imaging at E18. Analysis of EB3 trajectories exhibits increased EB3 speed in Taok2-deficient cells analyzed in culture or migrating in the developing cortex compared with WT or control cells (Fig. 2a–d and Videos 1 and 2), thus suggesting that microtubule dynamics are altered in the absence of Taok2.

To further test whether Taok2 affects microtubule dynamics, cortical neurons were transfected in utero with Taok2 shRNA or control shRNA, and the F-GFP plasmid (GAP43-GFP, to label the cell membrane) at E15 was dissociated and cultured at E17. Neurons were fixed after 2 days in culture and prepared for immunostaining. We found that neurites of cells expressing Taok2 shRNA had decreased levels of acetylated tubulin, a marker of stable microtubules, compared with control cells (Supplementary Fig. 6). Accordingly, it was shown that TAOK2 regulates microtubule organization and stability, promoting α-tubulin acetylation in Swiss 3T3 cells [17]. Moreover, JNK1 phosphorylation is regulated by Taok2 in rodent neurons [5] as well as human pluripotent stem cell-derived cortical neurons [18], and phosphorylated JNK1 (pJNK1) has been associated with stable microtubules [19]. Therefore, we performed immunostaining of acetylated tubulin and pJNK1 in DIV1-2 dissociated primary neurons obtained from KO and WT cortices. Since JNK1 phosphorylation is enriched in developing axons [20], we used cultured neurons during the initiation of polarization as a read-out system to test for levels of pJNK1. We found decreased pJNK1 signals in the longest process of Taok2 KO neurons compared to WT neurons (Fig. 2e, f). Concomitant with this result, the levels of acetylated tubulin were also reduced in KO cells compared with WT neurons (Fig. 2e, g). Finally, we detected that acetylated tubulin levels are decreased in lymphoblastoid cell lines (LCLs) derived from the A135P proband (Fig. 2j, k), suggesting fewer stable microtubules in LCL cells obtained from the A135P proband than in control cells from the unaffected father. However, we could not detect significant differences in pJNK1 levels in those cells (Fig. 2h, i). Of note, LCLs are made by LMP1 overactivation with Epstein‒Barr virus, which, in turn, activates TAK1 and MEK, which activate JNK to promote growth [21–24]. Therefore, changes in JNK phosphorylation due to TAOK2 may not be observed in these cells. Even though we could not detect differences in pJNK1 levels in LCLs, the expression of TAOK2αA135P and TAOK2βA135P variants in HEK cells differentially affected JNK1 phosphorylation, with TAOK2αA135P precluding JNK1 phosphorylation (Supplementary Fig. 7 and [8]). In line with these results, it was shown that TAOK2α, unlike TAOK2β, stimulates the JNK pathway in cell lines [25]. Altogether, our results show the important role of TAOK2 kinase activity in neuronal migration and highlight the cellular and mechanistic differences between the two TAOK2 isoforms.

### Taok2 modulates neuronal migration

**Fig. 3:**
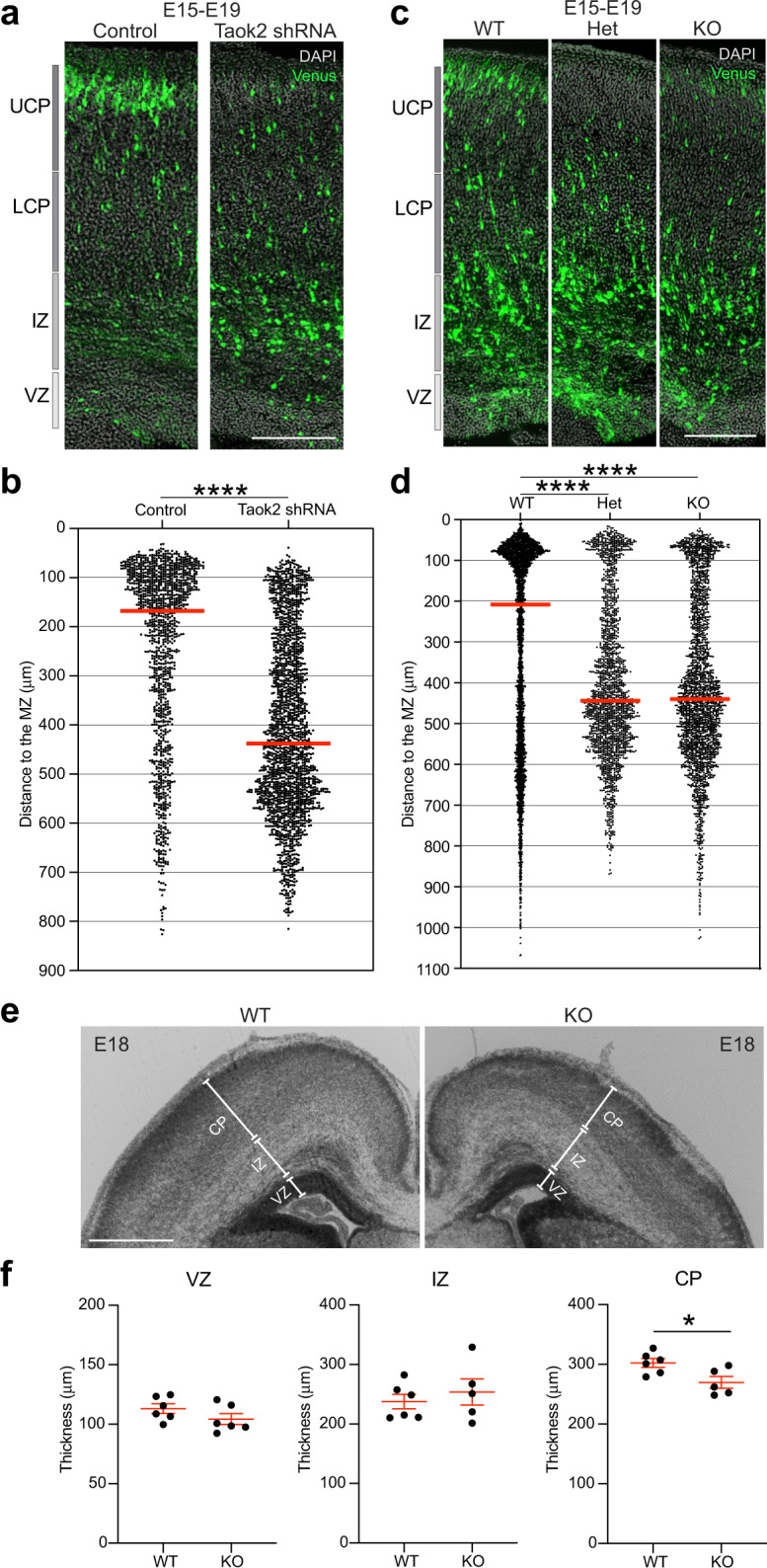
Taok2 deficiency affects neuronal migration and cortical thickness. **a** Taok2 downregulation disrupts neuronal migration in the developing cortex, with cells arrested in the IZ. **b** Quantification of cell distribution after Taok2 downregulation (****p < 0.0001 by unpaired *t-*test; control = 3 brains, Taok2 shRNA = 3 brains; median is represented by red line; see Supplementary Fig. 8a for individual brains). **c** Taok2 deficiency affects neuronal migration. **d** Quantification of the cell distribution of Venus-expressing cells in the developing cortex of WT, Het, and KO Taok2 mice (*p* < 0.0001 by one-way ANOVA, post hoc Dunnett’s multiple test ****p < 0.0001; WT = 4 brains, Het = 3 brains, KO = 3 brains; the median is represented by a red line; see Supplementary Fig. 8b for individual brains). The controls (WT) and KO from this experiment are the same as in Fig. 6d, given that this experiment was carried out at the same time. **e** Taok2-deficient brains reveal r**e**duced thickness of the CP at E18. **f** Quantification of the thickness of the VZ, the IZ and the CP in coronal sections of Taok2 WT and KO littermates (*p = 0.0241 by unpaired *t*-test WT = 6 brains, KO = 5–6 brains; values are the mean ± s.e.m.). Scale bar: 200 μm.

To further dissect the role of Taok2 in neuronal migration, we transfected cortices from WT, Het, and KO littermate embryos in utero with the Venus plasmid at E15 and analyzed the position of transfected cells in those cortices at E19. We simultaneously examined neurons at E19 that were transfected in utero at E15 using shRNA technology to silence Taok2. At E19, most control and WT-transfected neurons reached the CP (Fig. 3a–d and Supplementary Fig. 8 for the single brains analyzed). However, after acute Taok2 downregulation or in KO cortices, migrating neurons were held back in the IZ. Moreover, we measured the thickness of the ventricular zone (VZ), IZ, and CP in WT and KO developing cortices (E18) and found that only the CP thickness was reduced in the KO cortices compared with WT littermates (Fig. 3e, f). Our results show that Taok2 deficiency leads to disrupted cell positioning during cortex development, which affects CP thickness. However, these results do not distinguish between delayed neuronal migration or defects in radial glia architecture that might disrupt neuronal migration.

To discriminate between these two possibilities, we analyzed whether radial glia morphology is affected in Taok2 KO cortices. First, we immunolabeled coronal cortical slices from WT and KO brains harvested at E16 with antibodies against the radial glial marker Nestin. Nestin staining did not indicate any differences in the parallel arrangement of the glial cell processes in either condition (Supplementary Fig. 9a). We then immunolabeled coronal cortical slices from the same brains with antibodies against the extracellular matrix protein Reelin, synthesized and secreted by Cajal–Retzius cells in the marginal zone of the cortex, and could not observe any alterations in Reelin staining or composition in the marginal zone of those brains (Supplementary Fig. 9b). Moreover, in the VZ, we could not detect differences between WT and KO cortices regarding the expression pattern of beta-Catenin and the centrosome orientation in the apical domain of glial cells, as shown by Pericentrin staining (Supplementary Fig. 9c, d), suggesting a normal polarized morphology of radial glial cells in KO cortices. Next, we specifically downregulated Taok2 in early neurons using a neuron-specific promoter (pNeuroD) regulating Taok2 shRNA expression [26] and analyzed whether we could recapitulate the phenotype found in KO cortices or after using acute downregulation with Taok2 shRNA. Finally, with the in utero transfection of postmitotic cells at E15 using the pNeuroD promoter, we labeled cells that did not overlap with cells expressing the cell division marker Ki67 at E19 (Supplementary Fig. 9e, Frame 1 and 2). Importantly, we found that the expression of Taok2 shRNA in postmitotic cells changed the distribution of transfected cells in the developing cortex, with more cells in the IZ and fewer in the CP than in control-transfected cortices (Supplementary Fig. 9e, f). These results specifically support a neuron-specific cell-autonomous role of Taok2 in neuronal migration.

### Taok2 regulates the speed of migrating neurons

**Fig. 4:**
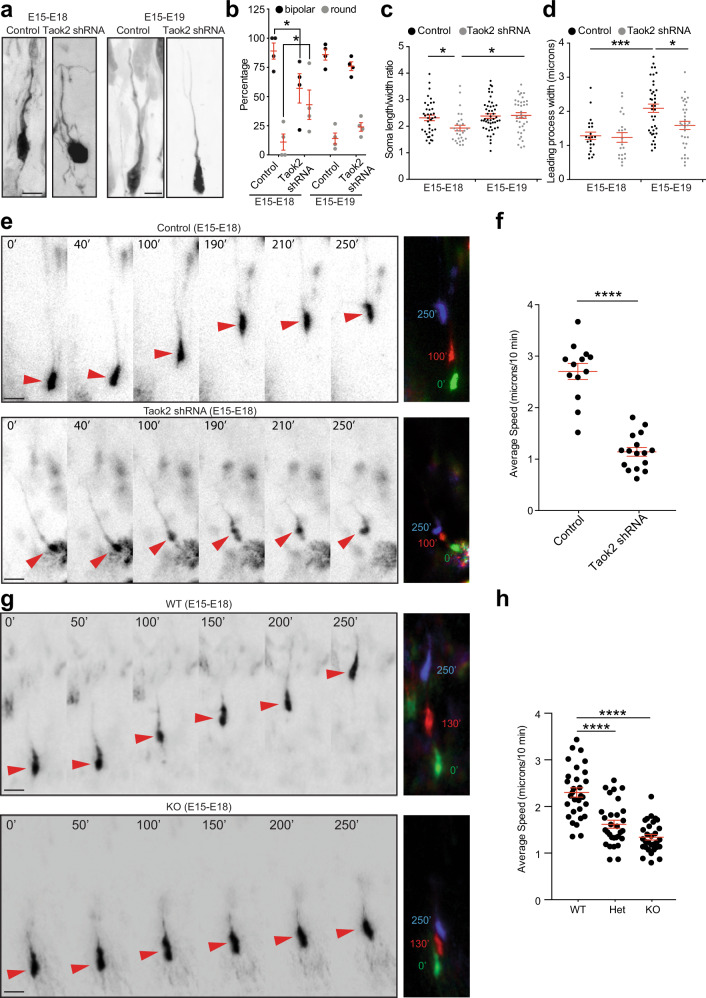
Taok2 deficiency decreases the speed of migrating neurons. **a** Cell morphology of migrating neurons in the CP of the developing cortex after expressing control shRNA or Taok2 shRNA. **b** Cell body morphology is transiently changed after Taok2 downregulation in migrating neurons in the CP. More round cells were found in E18 cortices after Taok2 downregulation (*p* < 0.0001, two-way ANOVA, post hoc Dunnett’s test *p < 0.05; control = 41 cells (E18) and 49 cells (E19), Taok2 shRNA = 29 cells (E18) and 39 cells (E19) for at least 3 brains per condition; values are the mean ± s.e.m.). **c** Quantification of soma length/width ratio of migrating neurons in the CP at E18 and E19, which were transfected in utero at E15 (*p* = 0.0340, two-way ANOVA, post hoc Tukey’s test *p < 0.05; control = 38 cells (E18) and 48 cells (E19), Taok2 shRNA = 28 cells (E18) and 37 cells (E19) for at least 3 brains per condition; values are the mean ± s.e.m.). **d** Quantification of the leading process width of migrating neurons in the CP at E18 and E19, which were transfected in utero at E15 (*p* < 0.0001, two-way ANOVA, post hoc Tukey’s test *p < 0.05 and ***p < 0.001; control = 22 cells (E18) and 40 cells (E19), Taok2 shRNA = 21 cells (E18) and 32 cells (E19) for at least 3 brains per condition; values are the mean ± s.e.m.). **e** Time-lapse analysis of migrating neurons in the CP of control shRNA- and Taok2 shRNA-transfected cells (red arrowheads). Right panel: three stacks superimposed (each color represents a different time point) show impaired speed of neuronal migration after acute Taok2 downregulation. **f** Quantification of neuronal migration speed in the developing cortex after Taok2 downregulation (****p < 0.0001 by *t-*test; control = 13 cells, Taok2 shRNA = 16 cells from slices of at least 3 brains per condition; values are the mean ± s.e.m.). **g** Time-lapse analysis of migrating neurons in the CP of WT and KO transfected cells (red arrowheads). Right panel: three stacks superimposed (each color represents a different time point) show impaired speed of neuronal migration in KO cells compared with WT-transfected cells. **h** Quantification of neuronal migration speed in the developing cortex of WT and KO animals (*p* < 0.0001 by one-way ANOVA; post hoc Tukey’s test ****p < 0.0001, WT = 5 brains, Het = 5, KO = 3 brains; values are mean ± s.e.m. WT = 30 cells, Het = 30 cells, KO = 30 cells from slices of different brains as indicated per condition; values are mean ± s.e.m.). Scale bar: 10 μm.

Our results in fixed tissue show impaired localization of migrating neurons in the developing cortex. However, these results do not discriminate between arrested neuronal migration and reduced migration speed in cells lacking Taok2. To directly test whether Taok2 deficiency decreased the rate of neuronal migration, we analyzed the cell morphology of migrating neurons in the CP as a snapshot of locomotion dynamics and performed time-lapse imaging in live cortex slices. After in utero transfection at E15, cells were obtained at either E18 or E19 for morphological analysis. Cells were classified as either bipolar or round, assuming bipolar cells reflect a more motile stage [27] (Fig. 4a).

Interestingly, only at E18 was a greater proportion of round cells present after Taok2 downregulation compared with control-transfected cells (Fig. 4a, b). This effect was transient since it was no longer detectable at E19. Measuring the length and width of the cell body corroborated this initial observation: the ratio of length/width was significantly decreased in Taok2 shRNA cells at E18 compared with control cells but not at E19 (Fig. 4c). Finally, the width of the leading process, proximal to the cell body, was measured assuming that a thicker leading process represents more motile cells during nucleokinesis [27]. Accordingly, our analysis revealed a thinner leading process after Taok2 downregulation at E19 compared with control-transfected cells (Fig. 4d). Overall, these results suggest that cells with acute loss of Taok2 might be less motile.

Based on our data from fixed tissue, we asked whether Taok2 downregulation decreases the speed of neuronal migration. To address this, cortices were electroporated in utero at E15 with Taok2 shRNA or control shRNA together with a Venus-expressing plasmid or with only a Venus-expressing plasmid when expressed in WT, Het, and KO littermates. Brains were harvested at E18 to prepare acute slices for time-lapse imaging of migrating cells within the CP. Supporting our previous observations in fixed tissue, the comparison between control/WT and Taok2-deficient cells revealed a significant decrease in migration speed after acute downregulation of Taok2 or in labeled neurons in Het and KO cortices compared with control or WT cortices, respectively (Fig. 4e–h and Video 3). Altogether, these results support the role of Taok2 in controlling the speed of neuronal migration.

**Fig. 5:**
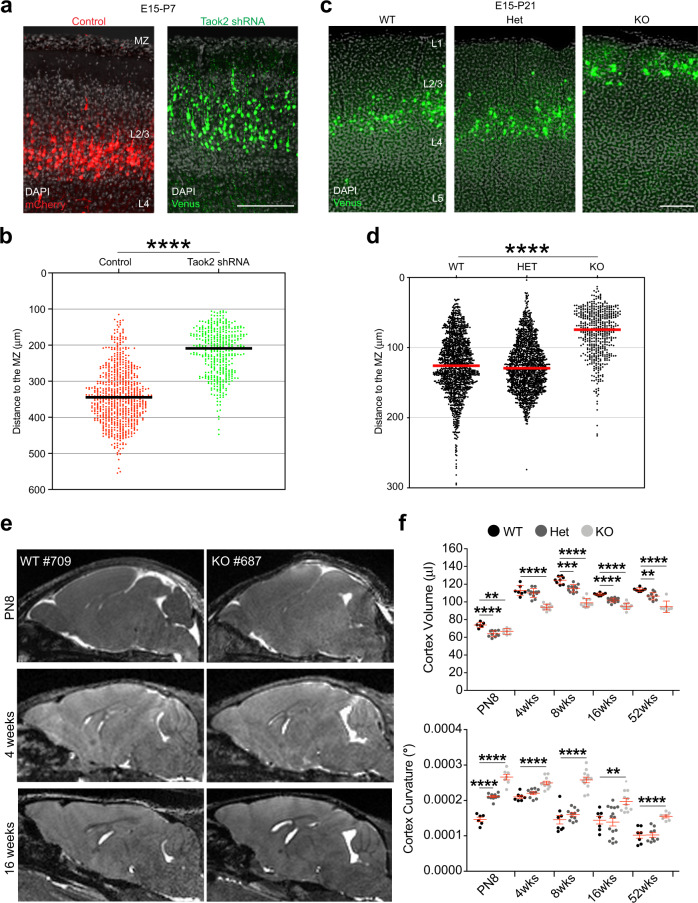
Taok2 deficiency affects cortex morphology. **a** Taok2 downregulation changes the position of upper-layer neurons at P7. Both brain hemispheres were electroporated in utero at E15 with control shRNA (mCherry) and Taok2 shRNA (Venus) on the contralateral sides. **b** Quantification of cell distribution at P7 (****p < 0.0001 by unpaired *t-*test; *n* = 6–7 brains per condition; median is represented by black line; see Supplementary Fig. 10a for individual brains). **c** Taok2 KO neurons, labeled in utero at E15, are localized more superficially in the upper cortex at postnatal day 21 than WT or Het neurons. **d** Quantification of cell distribution at P21 (*p* < 0.0001 by one-way ANOVA, post hoc Dunnett’s multiple test ****p < 0.0001; *n* = 3 brains per condition; median is represented by black line; see Supplementary Fig. 10b for individual brains). **e** Longitudinal MRI imaging of brains from WT and Taok2 KO mice shows morphological changes in the cortex in Taok2 KO brains compared with WT brains. **f** Quantification of cortex volume (upper panel) and cortex curvature (lower panel) at different time points (*p* < 0.0001 by one-way ANOVA, post hoc Dunnett’s test **p < 0.01, ***p < 0.001 and ****p < 0.0001, WT = 6–8 mice, Het = 9–15 mice, KO = 7–12 mice; values are the mean ± s.e.m.).

To further understand the outcome of delayed neuronal migration after acute Taok2 downregulation or in neurons lacking Taok2, we analyzed cortices at postnatal days 7 (P7) and 21 (P21), respectively. For acute Taok2 downregulation, mouse embryos were electroporated bilaterally in utero. The left hemisphere was electroporated with Taok2 shRNA and Venus plasmid, while the right hemisphere was electroporated with control shRNA together with mCherry plasmid as previously reported [5]. A comparison of the two hemispheres revealed that Taok2 shRNA-transfected neurons reached a higher position toward the pia than the control-transfected cells on the contralateral side (Fig. 5a, b and Supplementary Fig. 10a for the single brains analyzed). Notably, the populations of Taok2-downregulated neurons and contralateral control-transfected neurons both expressed Cux-1 (Supplementary Fig. 10b, c); thus, Taok2 deficiency does not affect layer identity.

Similarly, Venus-labeled neurons in the KO cortices showed a more superficial position at P21 compared with WT littermates (Fig. 5c, d and Supplementary Fig. 10d for the single brains analyzed). These results corroborate our initial observation in KO cortices, which showed that Cux-1-positive cells clustered toward the more superficial cortex [8], thus suggesting a cellular substrate for the reduction in cortex volume previously detected by us in Taok2 KO adult mice [8]. Accordingly, a longitudinal study using magnetic resonance imaging (MRI) revealed that the cortex of mice lacking Taok2 shows a significant reduction in volume throughout development concomitant with a higher cortex curvature—from early postnatal development until adulthood (Fig. 5e, f), a captivating trait of the human autistic disease within we did not observe any gender-specific alterations (Supplementary Fig. 10e, f). Taken together, these data with our preceding data on ASD-associated behavior in Taok2 KO mice [8], we have no indication of any gender-specific alterations in ASD-relevant phenotypes in Taok2 KO mice.

### Activation of the molecular Taok2-JNK1 signaling axis overcomes Taok2 deficiencies during neuronal migration

**Fig. 6:**
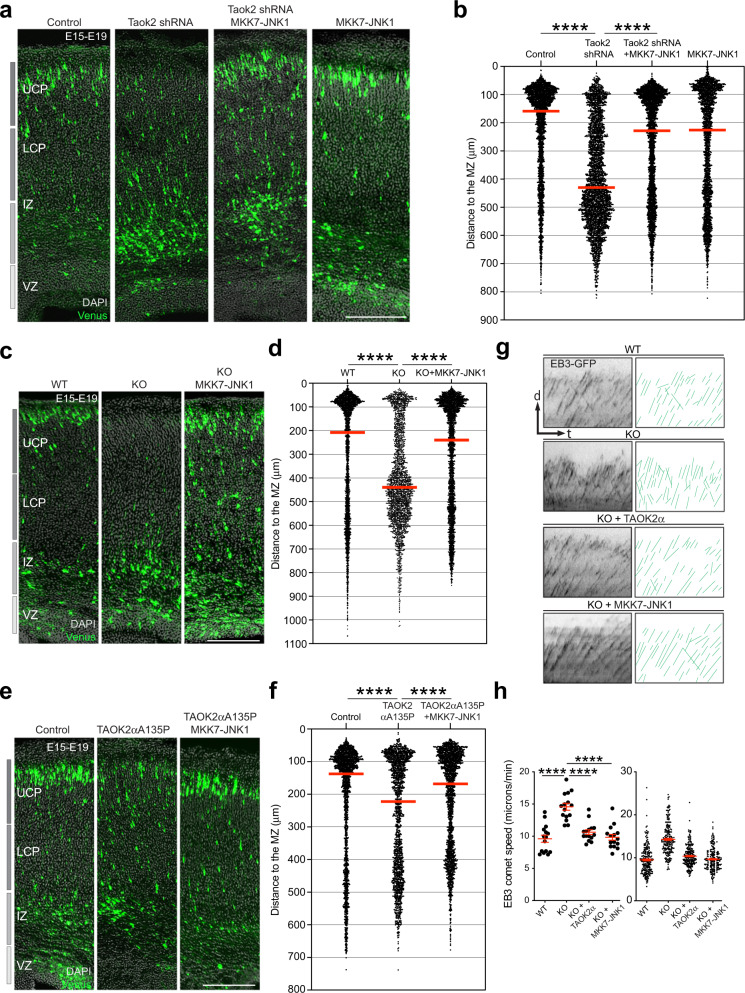
Activation of the molecular Taok2-JNK1 signaling axis overcomes Taok2 deficiencies during neuronal migration. **a** Expression of MKK7-JNK1 together with Taok2 shRNA ameliorates neuronal migration defects caused by Taok2 downregulation. **b** Quantification of cell distribution in the developing cortex shows a shift of neurons from the IZ toward the upper CP after MKK7-JNK1 expression compared with the cortices expressing only Taok2 shRNA (*p* < 0.0001 by one-way ANOVA, post hoc Dunnett’s multiple test ****p < 0.0001; Control = 5 brains, Taok2 shRNA = 5 brains, Taok2 shRNA + MKK7-JNK1 = 5 brains, MKK7 = 4 brains; median is represented by red line; see Supplementary Fig. 11a for individual brains). **c** Expression of MKK7-JNK1 ameliorates neuronal migration defects in Taok2 KO cortices. **d** Quantification of cell distribution in the developing cortex shows a shift of neurons from the IZ toward the UCP after MKK7-JNK1 expression compared with the untreated KO cortices (*p* < 0.0001 by one-way ANOVA, post hoc Dunnett’s multiple test ****p < 0.0001; WT = 4 brains, KO = 3 brains, KO + MKK7-JNK1 = 5 brains, MKK7 = 3 brains; median is represented by red line; see Supplementary Fig. 11b for individual brains). The controls (WT) and KO from this experiment are the same as in Fig. 3d, given that this experiment was carried out at the same time. **e** Expression of MKK7-JNK1 together with TAOK2αA135P ameliorates neuronal migration defects caused by the TAOK2αA135P mutation. **f** Quantification of cell distribution in the developing cortex shows a shift of neurons from the IZ toward the UCP after MKK7-JNK1 expression compared with the cortices expressing TAOK2αA135P (*p* < 0.0001 by one-way ANOVA, post hoc Dunnett’s multiple test ****p < 0.0001; Control = 4 brains, TAOK2αA135P = 4 brains, TAOK2αA135P + MKK7-JNK1 = 4 brains; median is represented by red line; see Supplementary Fig. 11c for individual brains). **g** Kymographs of EB3-GFP signals along neurites from WT (upper) and Taok2 KO cells (lower panels) in dissociated cultures. Kymograph analysis shows an overall change in the trajectories of EB3 comets (green lines) with steeper trajectories in Taok2 KO. Ectopic expression of TAOK2α and MKK7-JNK1 ameliorates increased microtubule dynamics in Taok2 KO cells. **h** Left graph: quantification of EB3 speed in the neurite shaft in dissociated cells. Taok2 KO cells show an increased EB3 speed that is restored after TAOK2α and MKK7-JNK1 expression. Right graph: all values for EB3 speed from the individual cells. (*p* < 0.0001 by one-way ANOVA, post hoc Dunnett’s multiple test ****p < 0.0001; *n* = 15 cells from three different cultures for each condition; values are the mean ± s.e.m.). Scale bar: 200 μm.

Given that we detected reduced acetylated microtubules and phosphorylated JNK1 in neurons lacking Taok2, we decided to test whether the molecular Taok2-JNK1 signaling axis previously described by us [5] might be a therapeutic avenue to ameliorate neuronal migration defects detected in the absence of Taok2. To this end, we introduced a constitutively active form of JNK1 (MKK7-JNK1) via in utero electroporation at E15 into cortices of either Taok2 KO mice or WT cortices expressing Taok2 shRNA or TAOK2αA135P and harvested transfected brains at E19. Introduction of MKK7-JNK1 ameliorated neuronal migration deficits in cortices expressing Taok2 shRNA (Fig. 6a, b and Supplementary Fig. 11a for the single brains analyzed), in cortices of Taok2 KO mice (Fig. 6c, d and Supplementary Fig. 11b for the single brains analyzed) and in cortices expressing TAOK2αA135P (Fig. 6e, f and Supplementary Fig. 11c for the single brains analyzed). To mechanistically evaluate whether these migration deficits downstream of TAOK2α convey regulation of microtubule dynamics involving JNK activity, we showed that ectopic WT TAOK2α expression and overexpression of MKK7-JNK1, respectively, plus co-transfection with fluorescence-labeled EB3 in Taok2 KO cells can ameliorate deficits of microtubule dynamics such as increased EB3 comet speed (Fig. 6g, h). Our data demonstrate that pJNK1 levels are decreased in Taok2-deficient cortical cells in vitro and that neuronal migration defects found in Taok2-deficient cortices or cortices expressing the human de novo mutation TAOK2αA135P could be rescued with the expression of constitutively active JNK1 in vivo, presumably through restoration of microtubule stability.

### TAOK2 ameliorates neuronal migration deficits in the 16p11.2 microdeletion mouse model

**Fig. 7:**
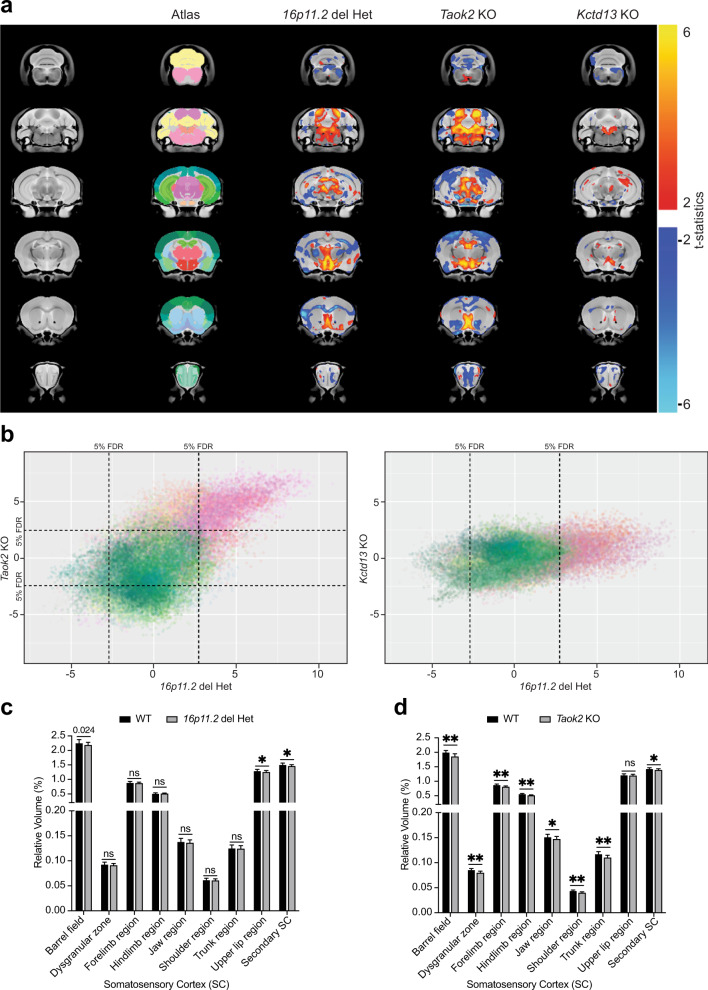
Alterations in brain anatomy in the 16p11.2 microdeletion mouse model resemble the phenotype of Taok2 KO mice but not of Kctd13 KO mice. **a** Het 16p11.2 microdeletion mice and Taok2 KO mice have altered brain morphology. A voxelwise analysis highlighting significant differences in relative volume (images show the lowest threshold of 5% false discovery rate (FDR) for Het 16p11.2 deletion mice, Taok2 KO mice and Kctd13 KO mice) throughout the brain between the respective WT and deletion mice. T-stats indicate positive or negative changes compared with the respective WT littermate brains. Taok2 KO mice but not 16p11.2 deletion or Kctd13 KO mice had increased absolute brain volume compared with WT mice (Taok2 WT = 16, KO = 23; 16p11.2 WT = 14, Het = 15; Kctd13 WT = 23, KO = 23 mice from three different cohorts, statistics by linear model. **b** Correlation analysis of relative volumes of brain regions in Het 16p11.2 deletion mice and in Taok2 KO mice revealed strong positive associations between Het 16p11.2 deletion and Taok2 KO mice but not between Het 16p11.2 deletion and Kctd13 KO mice; each point in the plot indicates a voxel in the brain, and the color indicates the anatomical area from which the voxel originated (colors from the Allen atlas, indicated in Column 2 of **a**). **c**, **d** The somatosensory cortex is particularly affected in Het 16p11.2 deletion mice and in Taok2 KO mice (statistics by linear model corrected for multiple comparisons using FDR; values are the mean ± S.D.; see Supplementary Table 1 for FDR and *p* values).

It was recently shown that neuronal migration is affected in organoids derived from patients lacking the 16p11.2 chromosomal region [28]. Moreover, brain size anomalies were reported in 16p11.2 CNV carriers with a reduction in cortex thickness [3], similar to the Taok2 KO mouse model [8]. Here, we compared the brain anatomy of the Het 16p11.2 deletion mouse model with the Taok2 KO and another ASD risk gene from the 16p11.2 chromosomal region, Kctd13 KO [10]. Using MRI of fixed brains from 8- to 10-week-old mice, we found that the brain anatomy of the Taok2 KO and 16p11.2 del Het mouse models shows a striking resemblance and more similarities than the Kctd13 KO mouse model when compared with the 16p11.2 del Het mouse model (Fig. 7a, b). The similarities between the Taok2 KO and 16p11.2 del Het mouse models are given in relation to the relative volume between subcortical and cortical brain areas (Supplementary Table 1). Specifically, the somatosensory cortex (SC), which we targeted to analyze neuronal migration in this study, showed reduced volume in the Taok2 KO and some regions of the SC of the 16p11.2 del Het mouse models compared with WT littermates (Fig. 7c, d). Differences detected in the 16p11.2 del Het mouse model compared to 16p11.2 del WT were not as drastic as in the Taok2 KO mouse model, probably due to the heterozygosis of the 16p11.2 del mouse model. Of note, we observed opposing effects of Taok2 loss on the volume of different brain regions in both Taok2 KO and Het 16p11.2 microdeletion mouse models for ASD. Hence, we correlated the effect of Taok2 loss on cortical (reduced volume) versus subcortical/mesencephalic (increased volume) brain regions with data on brain-region-specific Taok2 expression and phosphorylation [8]. Those brain regions with highly phosphorylated Taok2 (cortex, striatum, cerebellum) showed a reduction in volume when lacking Taok2, while in brain regions with the least phosphorylated Taok2 (hypothalamus, midbrain), loss of Taok2 led to an increase in volume. Our data suggest that Taok2 function in the brain is region specific and that the mechanism affecting brain volume that is prevalent in cortical areas might depend on Taok2 phosphorylation.

**Fig. 8:**
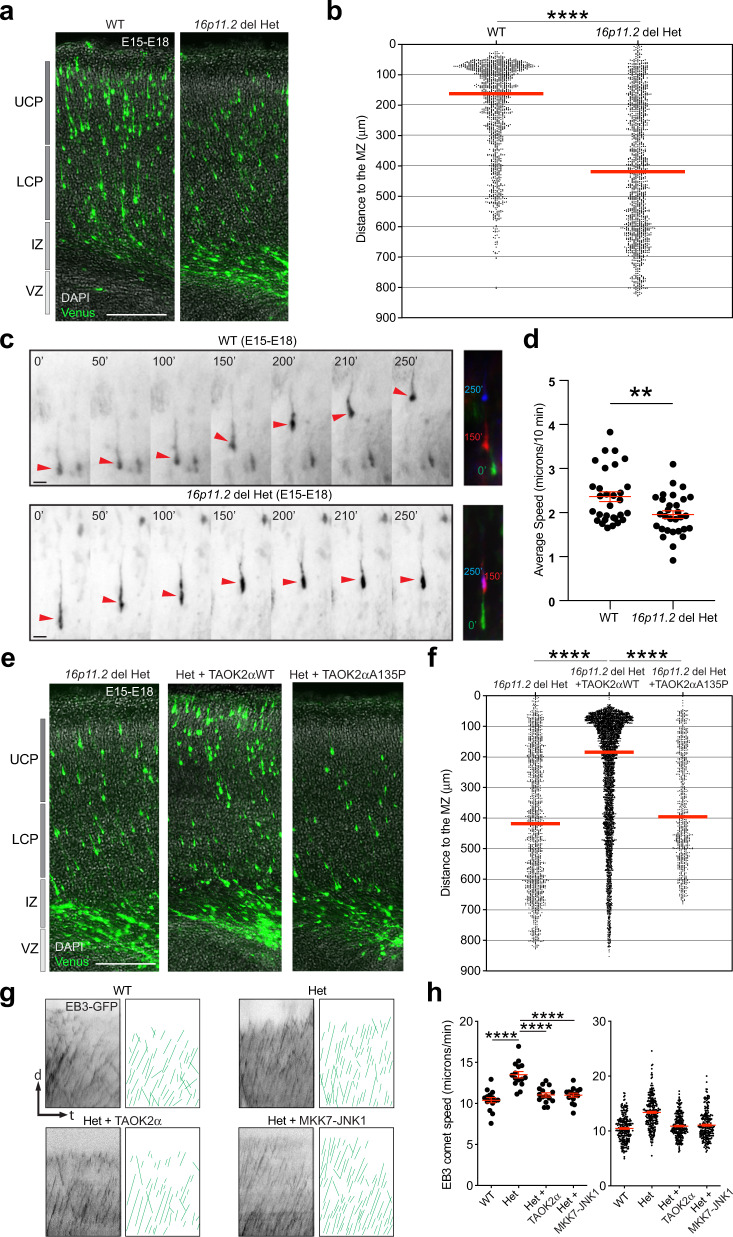
TAOK2 ameliorates neuronal migration deficits in the 16p11.2 microdeletion mouse model. **a** Loss of the ASD susceptibility gene locus 16p11.2 disrupts neuronal migration in the developing cortex with cells arrested in the IZ. **b** Quantification of cell distribution in the 16p11.2 deletion model (****p < 0.0001 by unpaired *t-*test; WT = 3 brains, 16p11.2 del Het = 4 brains; median is represented by red line; see Supplementary Fig. 12a for individual brains). **c** Time-lapse analysis of migrating neurons in the CP of WT and Het 16p11.2 deletion mice. Right panel: three stacks superimposed (each color represents a different time point) show reduced neuronal migration in 16p11.2 deletion mice. **d** Quantification of neuronal migration speed in the developing cortex in 16p11.2 deletion mice (**p = 0.0048 by unpaired *t-*test; WT = 30 cells, 16p11.2 del Het = 30 cells from slices of at least 3 brains per condition; values are the mean ± s.e.m.). **e** TAOK2αWT but not TAOK2αA135P rescues neuronal migration deficits in the Het 16p11.2 microdeletion mouse model. **f** Quantification of cell distribution in the developing cortex shows a shift of neurons from the IZ toward the UCP after TAOK2αWT expression compared with Het 16p11.2 cortices expressing only Venus or TAOK2αA135P (*p* < 0.0001 by one-way ANOVA, post hoc Dunnett’s multiple test ****p < 0.0001; 16p11.2 del Het = 4 brains, 16p11.2 del Het + TAOK2αWT = 6 brains, 16p11.2 del Het + TAOK2αA135P = 3 brains; see Supplementary Fig. 12a for individual brains; median is represented by red line). **g** Kymographs of EB3-GFP signals along neurites from WT (upper left panels) and Het 16p11.2 del cells (upper right panels) in dissociated cultures. Kymograph analysis shows an overall change in the trajectories of EB3 comets (green lines) with steeper trajectories in the Het 16p11.2 deletion. Ectopic expression of TAOK2α (lower left panels) and MKK7-JNK1 (lower right panels) ameliorates increased microtubule dynamics in Het 16p11.2 del cells. **h** Left graph: quantification of EB3 speed in the neurite shaft in dissociated cells. Het 16p11.2 del cells show an increased EB3 speed that is restored after TAOK2α and MKK7-JNK1 expression. Right graph: all values for EB3 speed from the individual cells. (*p* < 0.0001 by one-way ANOVA, post hoc Dunnett’s multiple test ****p < 0.0001; *n* = 15 cells from three different cultures for each condition; values are the mean ± s.e.m.). Scale bar: 200 μm.

Consequently, we decided to test whether the Het 16p11.2 microdeletion mouse shows neuronal migration defects in the developing cortex and whether TAOK2 could rescue those abnormalities. To this end, we transfected cortices from WT and Het 16p11.2 microdeletion littermates in utero at E15 with a Venus-expressing plasmid, with or without co-transfecting them with a TAOK2αA135P plasmid and analyzed the position of transfected cells in those cortices at E18–E19. Importantly, we found that Het 16p11.2 microdeletion cortices displayed altered cell positioning within the cortices of E18–E19 mice, with fewer transfected cells within the CP. In contrast, the number of transfected cells retained in the IZ was increased compared with that in WT littermates (Fig. 8a, b and Supplementary Fig. 12a for the single brains analyzed).

We then tested whether Het 16p11.2 microdeletion cortices show a decreased speed of neuronal migration based on our data from fixed tissue. To address this, cortices from WT and Het 16p11.2 microdeletion littermates were electroporated in utero with a Venus-expressing plasmid at E15, and brains were harvested at E18 to prepare acute slices for time-lapse imaging of migrating cells within the CP. Supporting our previous observations in fixed tissue, the comparison between WT and Het-expressing cells revealed a significant decrease in migration speed in labeled neurons in Het compared with WT cortices (Fig. 8c, d and Video 4). Altogether, these results show that Het 16p11.2 microdeletion affects neuronal migration.

Finally, we tested whether WT TAOK2α rescues neuronal migration deficits in the Het 16p11.2 microdeletion mouse model. Consequently, we electroporated cortices of Het 16p11.2 microdeletion mice at E15 with TAOK2αWT and Venus-expressing plasmids in utero. The cortices were harvested at E18–E19, fixed, and prepared for immunostaining. We found that the expression of TAOK2αWT in Het cortices increased the proportion of Venus-labeled cells within the CP compared to Het littermates expressing only Venus (Fig. 8e, f). On the other hand, the expression of TAOK2αA135P in the Het 16p11.2 microdeletion background did not overcome the neuronal migration deficit compared to that in Het littermates or Het 16p11.2 cells expressing TAOK2αWT (Fig. 8e, f and Supplementary Fig. 12a for the single brains analyzed). Accordingly, we found that this human mutation in TAOK2 impairs neuronal migration (Fig. 1a, b). To further evaluate the migration deficits mechanistically, we demonstrated that cortical neurons derived from the Het 16p11.2 microdeletion mouse model showed low levels of Taok2, reduced JNK1 phosphorylation and altered microtubule dynamics, including increased velocity of EB3 comets, in Het 16p11.2 cells (Fig. 8g, h and Supplementary Fig. 12b, c). Moreover, ectopic WT TAOK2α expression and overexpression of MKK7-JNK1 co-transfected with fluorescence-labeled EB3 in Het 16p11.2 del cells ameliorated deficits in microtubule dynamics, such as increased EB3 comet speed, thus regaining microtubule stability (Fig. 8g, h) and reversing the reduction in phosphorylated JNK-1 levels (Supplementary Fig. 12d, e). Altogether, our results show that the expression of TAOK2αWT ameliorates neuronal migration deficits in the Het 16p11.2 microdeletion mouse model supposedly by activation of JNK1 and stabilization of microtubules.

## Discussion

In the current report, we describe an isoform-specific functional role of TAOK2α in neuronal migration. The precise regulation of neuronal migration is critical for the proper development of brain architecture. Dysregulation of cortex development has been associated with ASD, among other neurodevelopment disorders [11]. We have previously shown that defective Nrp1-TAOK2-JNK1 signals reduced the formation of basal dendrites in the cortex [5], while TAOK2β affects isoform-specific synaptic morphology and connectivity through RhoA-dependent modulation of actin [8]. Our current work implicates activated JNK1 (pJNK1) as an effector of TAOK2α that modifies neuronal migration. JNKs are essential for many aspects of neuronal differentiation [29] and exert essential functions in neurodevelopment via microtubule dynamics [30]; thus, we propose that TAOK2α is a modulator of early developmental processes, such as neuronal migration, by affecting microtubules, while TAOK2β is implicated in later developmental processes, such as differentiation and connectivity, by affecting the actin cytoskeleton. Likewise, Jnk1 KO mice exhibit a progressive loss of microtubules within axons and dendrites [19]. Supporting our findings, it was shown that a dominant-negative form of JNK or the JNK inhibitor SP600125 slows neuronal migration [31]. However, an increased thickness of the E15/E18 telencephalon in Jnk1 KO mice was also reported. Importantly, two differently localized JNK1 forms were described: the cytoplasmic form, which delays neuronal migration, and the nuclear form of JNK1, which enhances migration [32]. In addition, it was reported that JNK1 works as a positive regulator of migration of interneurons, given that JNK antagonists resulted in reduced migration speed at E12 [33]. However, the identification of a protein candidate targeted by Taok2-JNK phosphorylation that can link the defective Nrp1-TAOK2-JNK1 signaling axis to the microtubule cytoskeleton to provoke deteriorated microtubule stability and dynamics during neuronal migration has not yet been identified. It was reported, however, that JNK1 regulates MT stability via phosphorylation of substrates such as SCG10 and MAP1B. SCG10 is a tubulin-sequestering protein that controls MT catastrophe events. SCG10 function is required for growth cone extension, and phosphorylation of SCG10 on serine 62 and serine 73 by JNK1 stabilizes MTs and promotes multipolar stage exit and neuronal migration rate [30, 32].

Recently, Taok2 has been identified as a molecular tether that links the endoplasmic reticulum (ER) with its extended network of membranes sheets and tubules to the microtubule cytoskeleton [12]. We demonstrated that a lack of Taok2 alters the distribution of the ER in the leading process of migrating neurons during cortical development. This is of particular interest since the connection of the ER to microtubules supports many cellular functions of the ER, including its motility and remodeling capacity [34] but also helps to generate specific membrane contact sites between the ER and other cell organelles, thus generating spatially discrete sections of the ER that can serve specific cellular functions [35, 36]. Importantly, an adverse effect of prolonged microtubule depolymerization by nocodazole on ER integrity and function has been described, causing a gradual collapse of peripheral ER into large cytoplasmic patches and membrane aggregates [37, 38]. Thus, the exact determination of whether the observed phenotype of altered distribution of ER protein in Taok2 KO neurons is due to deteriorated tethering of microtubule to the ER in the first place or whether the increase in microtubule dynamics in Taok2 KO cells due to the defective Nrp1-TAOK2-JNK1 signaling axis [5] causes the inaccurate distribution of ER remains unsolved. However, it was shown that the kinase activity of TAOK2 negatively regulates ER-microtubule tethering and that lack of TAOK2 and expression of a kinase-dead TAOK2 mutant have opposing effects on ER-microtubule tethering and dynamics in interphase and mitotic cells, providing strong evidence of bidirectional regulation of ER-microtubule tethering by Taok2 and evidence that the ER can directly affect microtubule dynamics [12]. Nonetheless, it is unquestionable that in highly polarized cells, such as bipolar neurons undergoing radial migration, an unaffected interplay between these two structural networks is crucial. Several lines of evidence have suggested that the establishment and maintenance of neuronal polarity as well as axonal development, integrity and function depend on local microtubule reorganization, on proper ER shape and on the extension of ER tubules into the axon orchestrated by motor proteins on microtubules; thus, all together on the correct interplay between microtubules and the ER [39–41]. The reciprocal nature of this interplay has been demonstrated since microtubules are essential for axonal ER tubule stabilization, while the ER is essential for stabilizing and organizing axonal microtubules. Therefore, the likely bidirectional instruction of ER-microtubule tethering by Taok2 indisputably makes this kinase an eminent and indispensable regulator of this network within the leading neurite of migrating bipolar neurons to instruct and/or maintain proper radial migration.

Whole-genome sequencing detects the genetic relevance of the 16p11.2 gene locus in ASD [1], facilitating brain imaging [42], behavioral [43], and network studies in this chromosomal region [44]. The TAOK2 gene is in the 16p11.2 genomic region and has known functions in central nervous system development [4–6]. Furthermore, 16p11.2 exhibits a spatiotemporal signature that is valid for most single genes encoded in this chromosomal region, such as TAOK2 [45]. Thus, for the 16p11.2 chromosomal region, the late mid-fetal and childhood phases are essential for proper connectivity in the human cortex. Furthermore, the frontal cortex, including motor and somatosensory areas, and the parietal, temporal and occipital cortices are highly susceptible to neurodevelopmental disorders, suggesting that neural disturbances in those regions contribute to neuropsychiatric disorders [45]. Importantly, we confirmed a spatiotemporal activity pattern for Taok2, as proposed for 16p11.2 and its single genes in humans [45].

Moreover, it was suggested that the prenatal phases in brain development could be significantly associated with ASD [46]. In particular, disturbances in the late mid-fetal stage affect almost all brain regions, excluding the cerebellum and the dorsolateral prefrontal cortex [46]. Therefore, our findings of delayed neuronal migration in upper-layer neurons from Taok2-deficient cortices support the hypothesis that late fetal neuronal differentiation in the cortex might affect connectivity in brain regions that could underlie the autistic phenotype. Finally, we showed cortical neuronal migration defects in the mouse model of Het 16p11.2 microdeletion that can be ameliorated by introducing functional TAOK2 into those cortices. Therefore, our data further support the role of TAOK2 during brain development and its implication in ASD etiology supposedly through impairing neuronal migration during the late mid-fetal phase of cortical development.

## Supplementary information

Video 1

Video 2

Video 3

Supplementary Materials and Methods

Supp Fig 1

Supp Fig 2

Supp Fig 3

Supp Fig 4

Supp Fig 5

Supp Fig 6

Supp Fig 7

Supp Fig 8

Supp Fig 9

Supp Fig 10

Supp Fig 11

Supp Fig 12

Supp. Table-1
