# Supplementary material for: TAOK2 rescues autism-linked developmental deficits in a 16p11.2 microdeletion mouse model
Source: Mol Psychiatry. 2022 Sep 19;27(11):4707–21. doi: 10.1038/s41380-022-01785-3 (PMC9734055; doi:10.1038/s41380-022-01785-3)
Supplement: Supplementary file 4 — Supplementary Materials and Methods [file 41380_2022_1785_MOESM4_ESM.docx]

**Materials and Methods**

***Animals***

*C57BL6/J Taok2* KO (*Taok2* -/-) mice were generated and described by Kapfhammer et al (1). Briefly, to generate a conditionally disrupted *Taok2* allele, lox P sites were introduced flanking exons 2 through 7 of the *Taok2* gene. Within intron 7, a neomycin resistance gene was inserted, flanked by lox P and frt sites. Upon crossing this line to mice expressing Flpe recombinase, the neomycin selectable marker was removed along with one of the lox P sites to generate the *Taok2tm1fl* allele. In the presence of Cre recombinase, the remaining lox P sites were recombined into a single lox P site, removing exons 2 through 7 and the translational start site to generate the *Taok2tm1Δ* allele. *16p11.2* Het microdeletion mice were purchased from Jackson Laboratories (Bar Harbor, ME, USA). Animals were bred, genotyped and housed at the Central Animal Facility at University Medical Center Hamburg-Eppendorf, Hamburg. All procedures received the approval of the Animal Research Ethics Board (AREB) and the Institutional Animal Care and Use committee of the City of Hamburg, Germany (N048/2013 and N007/2018 acc. to the Animal Care Act, §8 from May, 18th 2006). Two females were bred with one male per breeding cage. When maintaining a live *16p11.2* del colony, heterozygous *16p11.2* mice are bred with B6129SF1/J WT mice since mice homozygous for the deletion are embryonically lethal. Both female and male *Taok2* Het and KO mice and Het *16p11.2* deletion mice, respectively, were used for experiments. Genotypes were identified during breeding by PCR of ear notches or tail biopsies. 1- to 52-weeks old mice were used for live MRI imaging**.** To obtain cortical cultures, females were time-mated with males and males were removed when a plug was observed, indicating copulation. At, E17, E18 or E19, mothers were sacrificed and brains from embryos were collected. Animals of appropriate genotype were included.

***Magnetic Resonance Imaging of fixed brains***

8- to 10-weeks old mice were perfused with 4% PFA (with 2mM Prohance) in PBS. Mice were genotyped after perfusions and only mice with clear genotypes were used. Brains were left intact in the skull, and the zygomatic bone and muscles were removed. Brains were then left in 4% PFA (with 2mM Prohance) in PBS overnight and transferred to PBS (with 0.02% Sodium Azide and 2mM Prohance) and kept at 4°C. All mouse imaging was done at the Mouse Imaging Center (The Hospital for Sick Kids). A multi-channel 7.0 MRI scanner (Varian Inc., Palo Alto, CA) was used to image the brains within skulls. Sixteen custom-built solenoid coils were used to image the brains in parallel (2). Parameters for the anatomical MRI scans are as follows: T2-weighted, 3D fast spin-echo sequence, with a cylindrical acquisition of k-space, and with a TR of 350ms, and TEs of 12ms per echo for 6 echoes, field-of-view of 20x20x25 mm^3^ and matrix size = 504x504x630 giving an image with 0.040mm isotropic voxels. Total imaging time was 14h. To visualize and compare any changes in the mouse brains the images are linearly (6 parameter followed by a 12 parameter) and non-linearly registered together. All scans can then be resampled with the appropriate transform and averaged to create a population atlas representing the average anatomy of the study sample. The result of the registration is to have all scans deformed into alignment with each other in an unbiased fashion, allowing for the analysis of the deformations needed to take each individual mouse’s anatomy into the final atlas space to model how deformation fields relate to genotype (3, 4). The jacobian (a measure based on the deformation of each brain indicating expansion or contraction at that voxel) determinants of the deformation fields are then calculated by warping a pre-existing classified MRI atlas onto the population atlas. These measurements were then examined on a voxel-wise basis in order to localize the differences found within regions or across the brain, in a total of 182 different regions. Multiple comparisons in this study were controlled for using the false discovery rate (5).

***Magnetic resonance imaging (MRI) of live mice***

MRI was performed using a dedicated 7 Tesla small animal MRI (ClinScan, Bruker, Ettlingen, Germany) with a mouse head 4 element phased array receiver surface coil and a linear polarized rat body transmit coil. The animals were anesthetized with a gas mixture of oxygen and about 1% isoflurane. The oxygen was delivered with a flow rate of 0.5L/min and the isoflurane was applied via a vaporizer (Föhr Medical Instruments, Seeheim-Oberbeerbach, Germany). The animal's respiratory rate (about 100/min) was monitored using a small animal monitoring system (SA Instruments, Stony Brook, NY). Animal body temperature was not monitored, but a pad with circulating water of 37°C covering the back of the animal helped maintaining the body temperature during MRI. MRI for volumetric analysis was done using a 3D constructive interference steady state (CISS) sequence with echo time = 3.87 ms, repetition time = 7.74 ms, flip angle = 50°, readout bandwidth = 200 Hz/pixel, 4 averages, field of view = 16x16x14.4 mm^3^, matrix = 128x128x120, elliptical k-space sampling and 12:25 min scan time.

The curvature of the cortex VOIs (Volumes of interest) defined in the CISS (constructive interference steady state) data was also extracted using in house written *Matlab* code*.*

Briefly, a surface was fitted to the top points of the cortex VOI using the locally weighted scatter plot smoothing algorithm with the quadratic regression model (*loess*), then the mean and the Gaussian curvature was calculated and averaged over the midline cortex surface area.


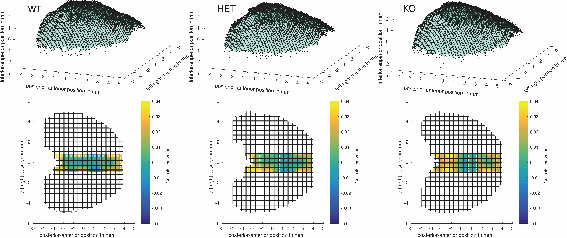


Surface fitted to upper points of the cortex VOI (black dots, upper row). The mean and the Gaussian curvature were calculated and averaged over the midline cortex surface area (lower row)

***shRNA and constructs***

The following constructs were used in these studies for transient expression in cell lines after liposomal transfection, for expression in dissociated neurons after Amaxa electroporation or for cortical expression after *in utero* electroporation, respectively: Wild-type TAOK2α (RC214297, NM_016151) and β isoforms (SC117141, NM_004783.2) were purchased (Origene, Rockville, MD). Human-derived mutations in TAOK2 were generated by site-directed mutagenesis (Agilent, Santa Clara, CA). cDNA sequences of WT or mutated TAOK2 were re-cloned into a recombinant adeno-associated virus (rAAV)-vector under control of a chicken β-actin promoter (pCAGIG). Silencing RNA shRNA ctrl and Taok2 shRNA were used as described before (6). A pSilencer vector containing a random sequence hairpin insert was used as a control for the shRNAs. Palmitoylated EGFP plasmid originates from Clontech, (Mountain View, CA, 6085-1). Flag-Jnk1a1 (pCDNA3) was a gift from Roger Davis (Addgene, Cambridge, MA; #13789). The MKK7-JNK1 (Plasmid 19726) was purchased from Addgene (Cambridge, MA). The Venus (pCAGIG) and mCherry (pCAGIG) plasmids were kindly provided by Dr. Z. Xie (Boston University). The F-GFP (pCAGIG-GAP 43 GFP) construct was a gift from Dr. A. Gartner (University of Leuven, Belgium). The Myc-TAOK2 (pCMV rat TAOK2) was kindly provided by Dr. M. H. Cobb (University of Texas Southwestern Medical Center). The EB3-mCherry construct (pCMV N2) was a gift from Dr. Frank Bradke (DZNE, Bonn, Germany) and the EB3-GFP construct (pCMV N1) was generously provided by Dr. Matthias Kneussel (ZMNH, Hamburg, Germany). Tubulin-GFP and ER-RFP were purchased (Addgene, Cambridge, MA; #56450 and #62236, respectively). Plasmids coding for neuronal-specific shRNA expression were kindly provided by Dr. K. Meletis (Department of Neuroscience, Karolinska Institute, Stockholm, Sweden) and produced as previously described by us (7).

***Antibodies***

The following antibodies were used in this studies: mouse anti-acetylated tubulin (Sigma, T7451; immunocytochemistry, 1:1000); goat anti-TAOK2 (Santa Cruz Biotechnology, K-16, western blot (WB), 1:2000; immunocytochemistry, 1:100); rabbit anti-TAOK2 beta (Synaptic Systems, 395 003, western blot (WB), 1:2500); rabbit anti-pTAOK2 (Ser 181; Santa Cruz Biotechnology, sc-135712, western blot (WB), 1:250; immunocytochemistry, 1:100); rabbit anti-active JNK (Promega, V7932; immunocytochemistry, 1:200); chicken anti-GFP (Aves Labs; GFP-1020, immunocytochemistry, 1:2000); rat anti-Ctip2 (Abcam, ab18465, immunocytochemistry, 1:100); rabbit anti-CUX1 (Santa Cruz Biotechnology, sc-13024, immunocytochemistry, 1:50); mouse anti-pan-JNK (BD Transduction Laboratories, 610627; western blot (WB), 1:500); rabbit anti-FAK (Santa Cruz Biotechnology, clone C-20, western blot (WB), 1:500); mouse anti-β-actin (Sigma, A5316, western blot (WB), 1:10.000) and mouse anti-GAPDH (Santa Cruz Biotechnology, sc-32233, western blot (WB), 1:5.000). Nuclei were visualized with Hoechst (Invitrogen, 33258; 1:10.000) and F-actin with phalloidin (Cytoskeleton, PHDR1; 1:500). Alexa conjugated secondary antibodies (Jackson Immunoresearch or Invitrogen, 1:1000) were applied for 1-2 hr at 25ºC.

***In utero* electroporation**

The Institutional Animal Care and Use Committee of the City of Hamburg, Germany approved all experiments (Approval N048/2013 and N007/2018 acc. to the Animal Care Act, §8 from May, 18th 2006). Time-pregnant C57BL/6J, *Taok2* or *16p11.2* del mice were given a pre-operative dose of buprenorphine (0.1 mg/kg body weight) by subcutaneous injections at least 30 min before surgery. Animals were then anesthetized using 2.5% isoflurane/O_2_ inhalation. Oxygen was delivered with a flow rate of 0.65L/min and together with isoflurane were applied via a vaporizer (Föhr Medical Instruments, Seeheim-Oberbeerbach, Germany). The uterine horns were exposed, and respective plasmids mixed with Fast Green (Sigma) were microinjected into the lateral ventricles of the embryos. Five current pulses (50ms pulse, 950ms interval; 35mV) were delivered across the heads of the embryos. Post-surgery, 2-3 drops of meloxicam (0.5 mg/kg body weight) were given orally through soft food for 96 hrs.

***Immunofluorescence***

*Dissociated neurons*: Neurons were fixed with 4% formaldehyde (FA) at 37 °C for 2 min followed by fixation for 3 min in methanol at -20 °C. After blocking in goat serum (Zymed), neurons were incubated with the primary antibodies. *Cortical sections*: Brains were removed and fixed overnight in 4% FA and thereafter transferred to 30% sucrose/PBS (4 °C, overnight). Brains were embedded in OCT compound and sectioned in a cryostat. The 20-30μm cryosections were incubated overnight at 4 °C with the primary antibodies.

***Confocal imaging***

Images were taken with a Zeiss LSM 510 confocal microscope. Z-series images were collected with 1μm steps. To perform 3D reconstructions on stacks of images of transfected cells, only Z sections in the same focal plane as GFP were used for analysis and for producing figures. 3D reconstructions and Z-stack analyses were produced using ImageJ software. The adjustment of brightness and contrast was performed on images.

***Organotypic slice cultures***

Mouse embryos were electroporated at embryonic day 15 (E15), and acute coronal brain slices (240μm) were prepared at E18 and E19 (at which time Taok2 was down-regulated via shRNA). For neuronal migration live imaging, slices were transferred onto slice culture inserts (Millicell) in cell culture dishes (35 × 10 mm; Corning) with Neurobasal medium (Invitrogen) containing the following: B27 (1%), glutamine (1%), penicillin/streptomycin (1%), horse serum (5%), and N2 (1%). Slices were used for imaging 1–2 h after cutting (incubated at 37°C in 5% CO_2_, for 1–2 d).

For EB3 comets live imaging in the slices, transfected slices were transferred to Ibidi glass bottomed dishes and imbedded with 1.5% collagen solution (Cultrex, R&D systems) with a final concentration of 1x DMEM F12. Once collagen solution was set, slices were covered with the aforementioned slice culture medium. EB-3 comets were imaged on an inverted Nikon microscope (TE 2000-S) with a 60× objective lens. During the time-lapse imaging, slices were kept in an acrylic chamber at 37°C in 5% CO_2_. We captured time-lapse images with a CoolSNAP EZ camera (Roper Scientific) using NIS-Elements software (Nikon).

***Cortical sections***

E15 *in utero* electroporated brains were removed at E18 or E19 and kept overnight in 4% PFA, which were then transferred to 30% sucrose/PBS (4°C, overnight) and embedded in OCT compound. Slices of 50μm thickness were sectioned using a cryostat and later incubated in PBS with Hoechst dye (1:10000, Invitrogen) and mounted with Fluoromount-G (SouthernBiotech).

***Cortical neuronal cultures and transfections***

Isolated cortices (from E18 embryos) were triturated in 1xHBSS (Invitrogen) after digested by papain and DNase for 10 min at 37°C (Worthington). Transfections were performed using the Amaxa nucleoporation system following the manufacturer’s manual. After electroporation, neurons were plated on poly-L-lysine coated glass coverslips (for immunostaining), on glass-bottomed dishes (ibidi, for live imaging) or tissue culture chambers (Sarstedt, for live imaging) in Neurobasal/B27 medium (Invitrogen), maintained in culture for 4~48 hours at 37°C with 5% CO_2_ before use.

***Lymphoblastoid cell lines (LCLs), SHSY5Y, and HEK293T cell lines***

LCLs were created using peripheral blood mononuclear cells isolated from the blood of probands and their families that were immortalized using the Epstein-Barr virus. Lymphoblastoid cell lines were cultured under standard suspension condition in RPMI 1640 media (with %15 FBS and 1mM sodium pyruvate). LCLs were cultured at 200.000 cells/ml in 5ml and media was doubled every 3-5 days until reaching 20ml. Cells were counted and 5 million cells were pelleted and lysed. Proteins from cell lysates were analyzed by BSA method (Thermo) for concentration determination and afterwards applied to immunoprecipitation or gel separation and western blotting. Proteins were separated on 12% SDS-polyacrylamide Tris-glycine gels at 100V and transferred to Immobilon-P PVDF membranes (Millipore) for Western Blot analysis. SHSY5Y neuroblastoma cells and HEK293T human embryonic kidney cells were originally purchased from ATCC (Manassas, VA, CRL-2266 and CRL-3216, respectively) and were generously provided by Dr. Matthias Kneussel (ZMNH, Hamburg, Germany). Both cell lines were transfected according to the manufactorer’s protocol using Lipofectamine^TM^ LTX Reagent with PLUS Reagent (ThermoFisher Scientific, Austin, TX, 15338100).

***Amaxa Electroporation***

Amaxa electroporation (Lonza) was carried out according to the manufacturer's protocol and described elsewhere. In detail, 5x10^6^ cells were resuspended in 100µl Nucleofector solution with a total of 3µg DNA or the indicated amount of shRNA added. Rat hippocampal neurons and mouse cortical neurons were transfected using the appropriate Nucleofector kit using program G-013 or O-005, respectively. Transfected neurons were plated on glass coverslips in 24-well chambers (50,000 cells/well), pre-coated with 0.1mg/mL Poly-D-Lysine (BD Sciences)/3.3ug/mL Laminin (Sigma) for immunocytochemical analysis or on pre-coated 4-well LabTek chamber slides (300,000 cells/chamber, Sarstedt, Nürnbrecht, Germany) for time-lapse life imaging.

***Time-lapse imaging***

GFP- and mCherry-positive cells were imaged on an inverted Nikon microscope (TE 2000-S) with a 20× objective lens [numerical aperture (NA) 0.45]. During the time-lapse imaging, slices were kept in an acrylic chamber at 37°C in 5% CO_2_. We captured time-lapse images with a CoolSNAP EZ camera (Roper Scientific) using NIS-Elements software (Nikon).

***Analysis of neuronal migration and migration velocity***

For quantification of neuronal migration. Two-dimensional coordinates of neurons were captured as ROIs with the point selection tool of ImageJ. The coordinates of the cortex surface were captured as ROIs with the segmented line tool of ImageJ. The shortest distance of a neuron to the cortex surface was calculated in a self-written script in Rstudio. Visual representation of data was performed in GraphPad Prism.

The neuronal migration velocity was measured using a plugin for ImageJ (Mouse Tracker, programmed by P. Malatesta, IST Genova) that allows tracking the cell position over time. Using the coordinates obtained with ImageJ, the velocity was calculated with Excel (Microsoft).

***EB3 comets quantifications***

For measuring the average speed of EB3 comets in the neurites, time-lapses of mouse cortical neurons (stage 2 and stage 3) co-transfected with EB3-GFP were used. For measuring the average speed of EB3 comets in transfected cells in acute slices, time-lapses of neurons co-transfected with EB3-GFP were used. Only in case of neuroblastoma cells (SHSY5Y), cells were co-transfected with EB3-mCherry and either ctrl or one of the TAOK2 variants. Lines were drawn along the length of each neurite shaft to generate kymographs (with a line width of 1). From the kymographs, the slope of each recognizable EB3 comet was measured. Comets were tracked via ImageJ’s TrackMate (3.7.0) (8). Tracking was performed in semi-automatic mode. Imaging and analysis were done blinded, cells which showed mCherry clusters were excluded from the analysis. Data were represented as average speed of EB3 comets (µm/min).

**Age dependent brain lysates**

Total brains (E11.5 and E13.5) or cortices from embryonal 15.5-days old to postnatal 22-days old C57BL/6 mice were dissected and homogenized in 20x vol/weight of sterile-filtered RIPA buffer (50mM Tris-HCl pH 7.4, 150mM NaCl, 1mM EGTA, 1% NP-40, 0.25% sodium deoxycholate, 10mM Na_3_VO_4_, 10mM NaF, containing proteinase inhibitors (Roche, Basel, Switzerland). To clear lysates, they were centrifuged at 20.500 x g for 10 min at 4°C. Supernatants were analyzed by BCA method (Thermo Scientific) for concentration determination and afterwards either applied to immunoprecipitation or to SDS gel separation and western blotting directly. Phospho Taok2 was detected from immunopreciptations while total Taok2, Taok2β and β-Actin were detected in lysates. To demonstrate that equal amounts of total proteins were loaded, Amido Black stainings (0.1% Amido Black, 50% EtOH, 10% HAc) were carried out before PVDF membrane blocking.

**Immunoprecipitation**

400μg of total lysates for Taok2 immunoprecipitation were incubated overnight at 4°C with 2μg of pre-conjugated polyclonal TAOK2 antibody (Santa Cruz). Antibodies were conjugated before to 15μl of magnetic Dynabeads™ Protein G (Invitrogen) for 2hrs at RT. A magnet was used to collect the bound immune complexes, followed by 5 washes in sterile-filtered RIPA buffer. After the final wash, supernatants were completely removed and beads were eluted in 2x SDS-PAGE sample buffer, boiled for 5 min at 95 °C and separated on 4-20% polyacrylamide gradient gels (Invitrogen).

**Statistics**

Statistical analyses were performed using GraphPad Prism 8. Data were tested for significant differences (*p < 0.05, **p < 0.01 and ***p < 0.001) using unpaired t-test, non-parametric one-, or two-way repeated-measures ANOVA. To compare variances either F-test or Bartlett's test were used. As post-hoc test for multiple comparison either Dunnett’s, Sidak’s or Tukey’s were used, depending on the comparison. No statistical measures were used to estimate the sample size, since the effect size was unknown. No randomization was used for the assignment of animals to groups, since the assignment was based on genotype.

**References**

1. Kapfhamer D, Taylor S, Zou ME, Lim JP, Kharazia V, Heberlein U. Taok2 controls behavioral response to ethanol in mice. Genes, brain, and behavior. 2013;12(1):87-97.

2. Bock M, Umathum R, Zuehlsdorff S, Volz S, Fink C, Hallscheidt P, et al. Interventional magnetic resonance imaging: an alternative to image guidance with ionising radiation. Radiation protection dosimetry. 2005;117(1-3):74-8.

3. Nieman BJ, Flenniken AM, Adamson SL, Henkelman RM, Sled JG. Anatomical phenotyping in the brain and skull of a mutant mouse by magnetic resonance imaging and computed tomography. Physiological genomics. 2006;24(2):154-62.

4. Lerch JP, Pruessner J, Zijdenbos AP, Collins DL, Teipel SJ, Hampel H, et al. Automated cortical thickness measurements from MRI can accurately separate Alzheimer's patients from normal elderly controls. Neurobiology of aging. 2008;29(1):23-30.

5. Genovese CR, Lazar NA, Nichols T. Thresholding of statistical maps in functional neuroimaging using the false discovery rate. NeuroImage. 2002;15(4):870-8.

6. de Anda FC, Rosario AL, Durak O, Tran T, Graff J, Meletis K, et al. Autism spectrum disorder susceptibility gene TAOK2 affects basal dendrite formation in the neocortex. Nat Neurosci. 2012;15(7):1022-31.

7. de Anda FC, Meletis K, Ge X, Rei D, Tsai LH. Centrosome motility is essential for initial axon formation in the neocortex. J Neurosci. 2010;30(31):10391-406.

8. Tinevez JY, Perry N, Schindelin J, Hoopes GM, Reynolds GD, Laplantine E, et al. TrackMate: An open and extensible platform for single-particle tracking. Methods. 2017;115:80-90.

**Supplementary Figure 1.** **Expression profile of pTaok2 and Taok2 during cortex development.** (**a, b**) Immunoprecipitations (a) and Western blot analysis of lysates (b) showing pTaok2 levels and the expression pattern of total Taok2 and Taok2β throughout cortical development. Taok2 expression increases considerably at perinatal (E19) and postnatal time points, meanwhile pTaok2 peaks at postnatal day 1 and 4 (PN1, 4). (**c**) Amido Black staining was used to demonstrate equal loading of total proteins since actin expression also increased with time. (**d**) Quantification of pTaok2 (left), total Taok2 (middle) and Taok2β (right) at different time points. Protein levels were normalized to the first time point (E11; pTaok2 = p<0.0025 by one-way ANOVA, *post hoc* Dunnett’s test *p<0.05, **p<0.01, ***p<0.001; total Taok2 = p<0.0026 by one-way ANOVA, *post hoc* Dunnett’s test *p<0.05, **p<0.01, ***p<0.001; Taok2β = p<0.0001 by one-way ANOVA, *post hoc* Dunnett’s test *p<0.05, ***p<0.001, ****p<0.0001; n = 5-8; values are mean ± s.e.m.

**Supplementary Figure 2. Position of deep layer neurons is not affected in *Taok2* Het or KO animals compared with WT littermates.** (**a**) Distribution of Ctip positive cells in deep cortex layers from WT and KO brains. (**b**) Quantification of thickness of Ctip positive layer in cortices from WT, Het, and KO animals (3 and 16 weeks old; p<0.0001 by one-way ANOVA, *post hoc* Sidak’s test). n = 3-9 brains per condition; values are mean ± s.e.m. Scale bar: 200 μm

**Supplementary Figure 3 (**Related to Figure 1). (**a**, **b**) Distribution of Venus positive migrating neurons from the independent brains analyzed.

**Supplementary Figure 4. TAOK2α localizes with microtubules in cultured neurons.** (**a**) TAOK2α co-localizes with microtubules (acetylated tubulin, upper panel) but not with F-actin (phalloidin, lower panel). (**b**) TAOK2β (green) does not co-localize with acetylated microtubules (red) but accumulates in filopodia (green arrow). (**c**) Upper graph: profile of the acetylated tubulin and TAOK2α signal from (a; line and arrowhead from the inset). Lower graph: profile of the acetylated tubulin and TAOK2β signal from (b; line and arrowhead from the inset). (**d**) Tethering of microtubules to the endoplasmic reticulum is deteriorated in migrating neurons in *Taok2* KO mice. (**e**) Quantification of ER distance to neurite base (left), ER-positive area (middle) and ratio of Tubulin- to ER-positive area (right) in the leading process of migrating neurons in the CP at E18 which were *in utero* transfected with ER-RFP and Tubulin-GFP at E15 (**p<0.01 and ****p<0.0001 by unpaired t-test; WT = 65 cells, TaoK2 KO = 66 cells from three different brains; values are mean ± s.e.m.). Scale bar 10 µm

**Supplementary Figure 5.** **TAOK2α, but not TAOK2β, modulates microtubule dynamics.** (**a-c**) Left panel: Representative SHSY5Y cells expressing EB3-mCherry (**a**), EB3-mCherry+TAOK2α (**b**), and EB3-mCherry+TAOK2αA135P (**c**). Right panel: Kymographs from green line in left panel show the EB3 trajectories for each condition. (**d**) Left graph: Quantification of EB3 speed in SHSY5Y cells expressing a control plasmid, WT TAOK2α or TAOK2αA135P, WT TAOK2β or TAOK2βA135P. Right graph: all values for the EB3 speed from all condition tested. (****p<0.0001 by one-way ANOVA, *post hoc* Dunnett’s test ***p<0.001; n = 15 cells per condition; values are mean ± s.e.m). Scale bar: 10 μm

**Supplementary Figure 6. Taok2 down-regulation decreases acetylated tubulin.** (**a**) Taok2 down-regulation (lower panel) decreased acetylated tubulin levels in cultured neurons compared with control-transfected cells (upper panel). (**b**) Quantification of acetylated tubulin after Taok2 down-regulation in cultured neurons (****p<0.0001 by t-test; control = 23 cells, Taok2 shRNA = 25 cells from three different cultures; values are mean ± s.e.m). Scale bar: 10 μm

**Supplementary Figure 7.**  **TAOK2α, but not TAOK2β, affects phosphorylation of JNK1.** (**a**) Western blot analysis of HEK cell lysates showing pTaok2 and pJNK1 levels after expressing TAOK2α, TAOK2αA135P, TAOK2β, TAOK2βA135P, and TAOK2βP1022. (**b**) Quantification of pJNK1 (****p<0.0001 by one-way ANOVA, *post hoc* Dunnett’s test ****p<0.0001; n = 5-7; values are mean ± s.e.m).

**Supplementary Figure 8** (Related to Figure 3). (**a**, **b**) Distribution of Venus positive migrating neurons from the independent brains analyzed.

**Supplementary Figure 9. Neuronal migration disruption in Taok2 deficient cortices is neuronal specific.** (**a**) Radial glia organization is not affected in *Taok2* KO cortices (black arrowheads, right panel) compared with WT cortices (black arrowheads, left panel). (**b**) Reelin in the marginal zone is not affected in *Taok2* KO cortices (right panel) compared with WT cortices (left panel). (**c**) β-catenin in the ventricular zone is not affected in *Taok2* KO cortices (right panel) compared with WT cortices (left panel). (**d**) Centrosome organization in the ventricular zone is not affected in *Taok2* KO cortices (right panel) compared with WT cortices (left panel). (**e**) Specific neuronal Taok2 down-regulation disrupts neuronal migration. Expression of GFP under neuronal promoter pNeuroD is specific for cells, which do not express the cell cycle marker Ki67. Taok2 down-regulation under the neuronal pNeuroD promoter disrupts cell positioning with cells stuck in the IZ (**f**) Quantification of cell distribution in the developing cortex shows cells piled up in the IZ after specific neuronal downregulation of Taok2 compared with the control transfected cortices (***p<0.001 by unpaired t test; 3 brains per condition (lower panel); median is represented by red line). Scale bar: 100 μm

**Supplementary Figure 10 (Related to Figure 5). Taok2 down-regulation does not change layer II identity.** (**a**) Distribution of Venus positive migrating neurons from the independent brains analyzed (Figure 5b). (**b**) Taok2 deficient upper layer neurons (Venus + cells, right panel) express Cux-1 similar to control transfected cell (mCherry + cells, left panel). (**c**) Quantification of Cux-1 + cells expressing Taok2 shRNA and control shRNA (p=0.3545 by unpaired t test; 6-7 brains per condition; values are mean ± s.e.m). (**d**) Distribution of Venus transfected neurons from the independent brains analyzed (Figure 5d). (**e, f**) Longitudinal MRI imaging of brains from WT and *Taok2* KO mice show no gender-specific alterations in cortex volume at 8- and 16-weeks of age, respectively. (p<0.0001 by one-way ANOVA, *post hoc* Dunnett’s test ***p<0.001 and ****p<0.0001, Males: WT = 3-4 mice, Het = 8 mice, KO=7-8 mice; Females: WT = 4 mice, Het = 3-7 mice, KO=3-5 mice; values are mean ± s.e.m). Scale bar: 100 µm

**Supplementary Figure 11** (Related to Figure 6). Distribution of Venus positive migrating neurons from the independent brains analyzed.

**Supplementary Figure 12**. (**a**) Distribution of Venus positive migrating neurons from the independent brains analyzed (Related to Figure 8). (**b**) Western blot analysis of cortical neurons derived from WT or Het *16p11.2* del mice show reduced Taok2 and pJNK1 expression. (**c**) Quantification of normalized Taok2 and pJNK1 (**p<0.01 and ****p<0.0001 by unpaired t-test; n = 3; values are mean ± s.e.m). (**d**) Cultured cortical neurons (2 DIV) from WT and *Het 16p11.2* del brains (upper), Amaxa-transfected with EB3-GFP before plating, show less pJNK1 (red) expression in Het *16p11.2* del cells compared with WT cells (red arrowheads). Ectopic WT TAOK2α and MKK7-JNK1 expression (lower panel), respectively, co-transfected with EB3-GFP in Het *16p11.2* del cells, restore reduced levels of phosphorylated JNK-1. (**e**) Quantification of pJNK1 content in longest neurite. Het *16p11.2* del cells have reduced pJNK1 expression levels and ectopic expression of WT TAOK2α or MKK7-JNK1 can restore pJNK1 (p<0.0001 by one-way ANOVA, *post hoc* Dunnett’s test ****p<0.0001; WT = 31cells, Het *16p11.2* del = 43 cells, Het *16p11.2* del + TAOK2α = 27 cells and Het *16p11.2* del + MKK7-JNK1 = 26 cells from three cultures; values are mean ± s.e.m).

**Video 1. Dissociated cortical neurons lacking Taok2 have increased EB3 speed.** Fluorescence imaging was performed on an inverted Nikon microscope (Eclipse, Ti) with a 60x objective (NA 1.4). Duration of time-lapse imaging: 5 min acquiring images every 2 sec.

**Video 2. Migrating neurons *in situ* lacking Taok2 have increased EB3 speed.** Fluorescence imaging was performed on an inverted Nikon microscope (Eclipse, Ti) with a 60x objective (NA 1.4). Duration of time-lapse imaging: 5 min acquiring images every 2 sec.

**Video 3. Neuronal migration speed is reduced after Taok2 down-regulation *in situ*.** Fluorescence imaging was performed on an inverted Nikon microscope (Eclipse, Ti) with a 60x objective (NA 1.4). Duration of time-lapse imaging: 250 min acquiring images every 10 min.
