# Supplementary figures and images for: TAOK2 rescues autism-linked developmental deficits in a 16p11.2 microdeletion mouse model
Source: Mol Psychiatry. 2022 Sep 19;27(11):4707–21. doi: 10.1038/s41380-022-01785-3 (PMC9734055; doi:10.1038/s41380-022-01785-3)

**a**

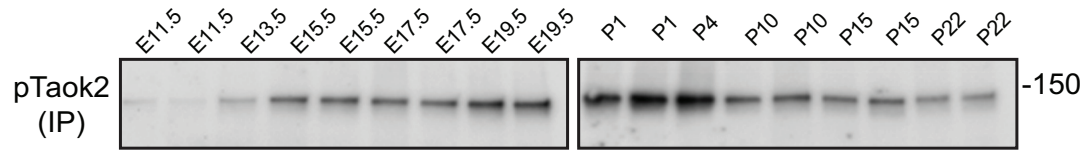

**b**

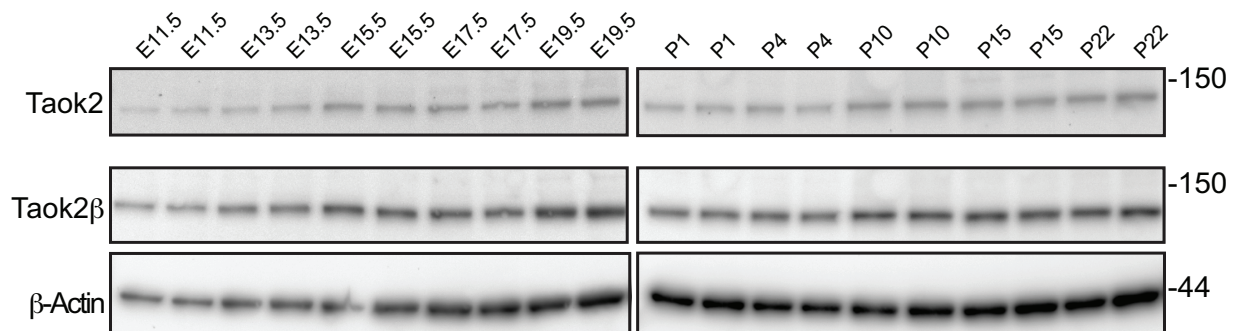

**c**

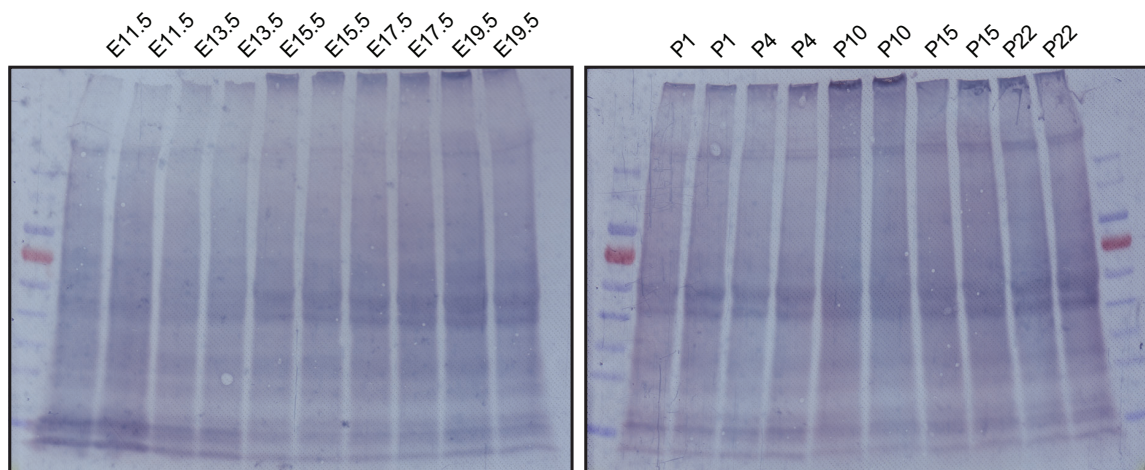

**d**

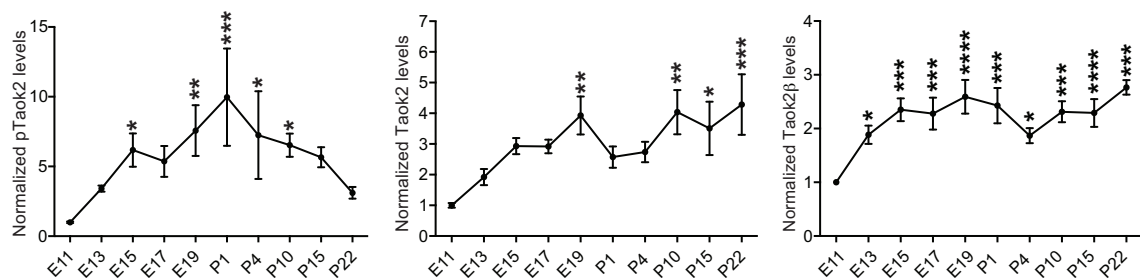

Supplement: Supplementary file 5 — Supp Fig 1 [file 41380_2022_1785_MOESM5_ESM.pdf]

**a**

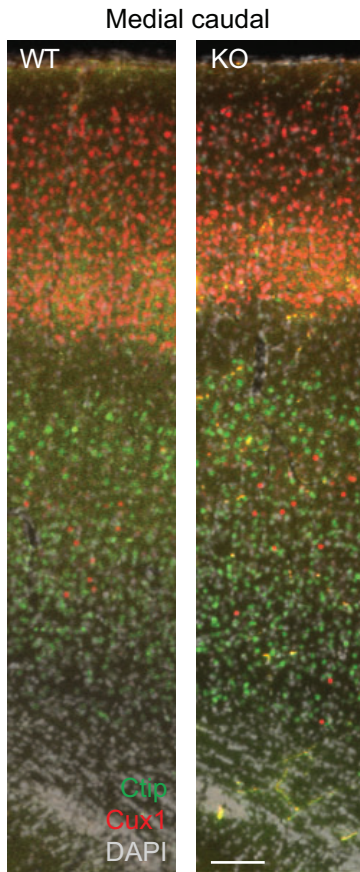

**b**

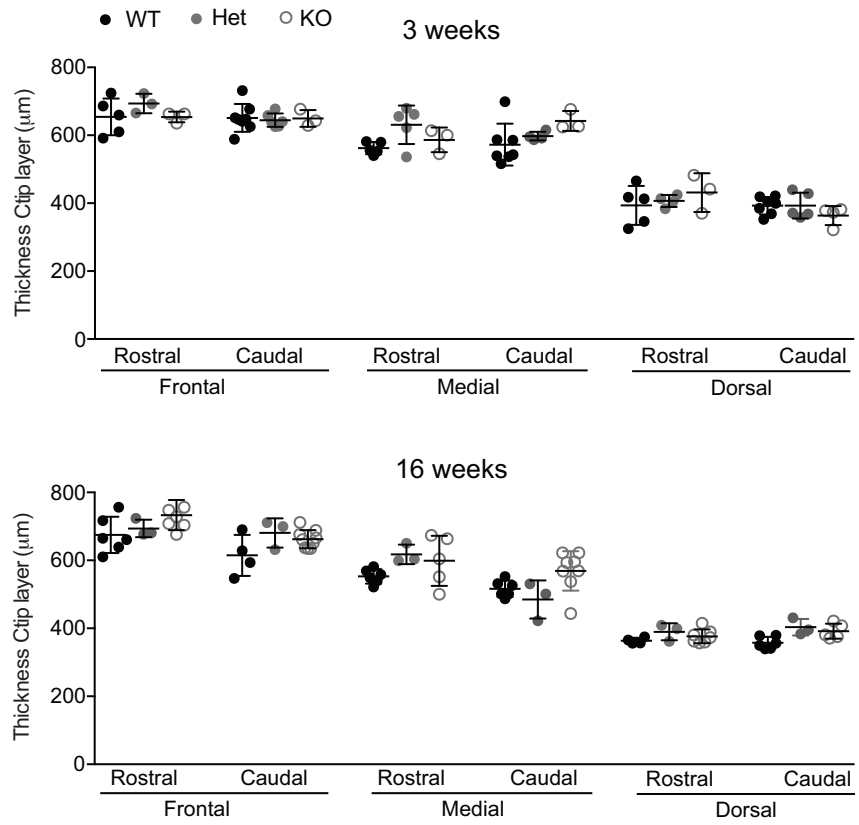

Supplement: Supplementary file 6 — Supp Fig 2 [file 41380_2022_1785_MOESM6_ESM.pdf]

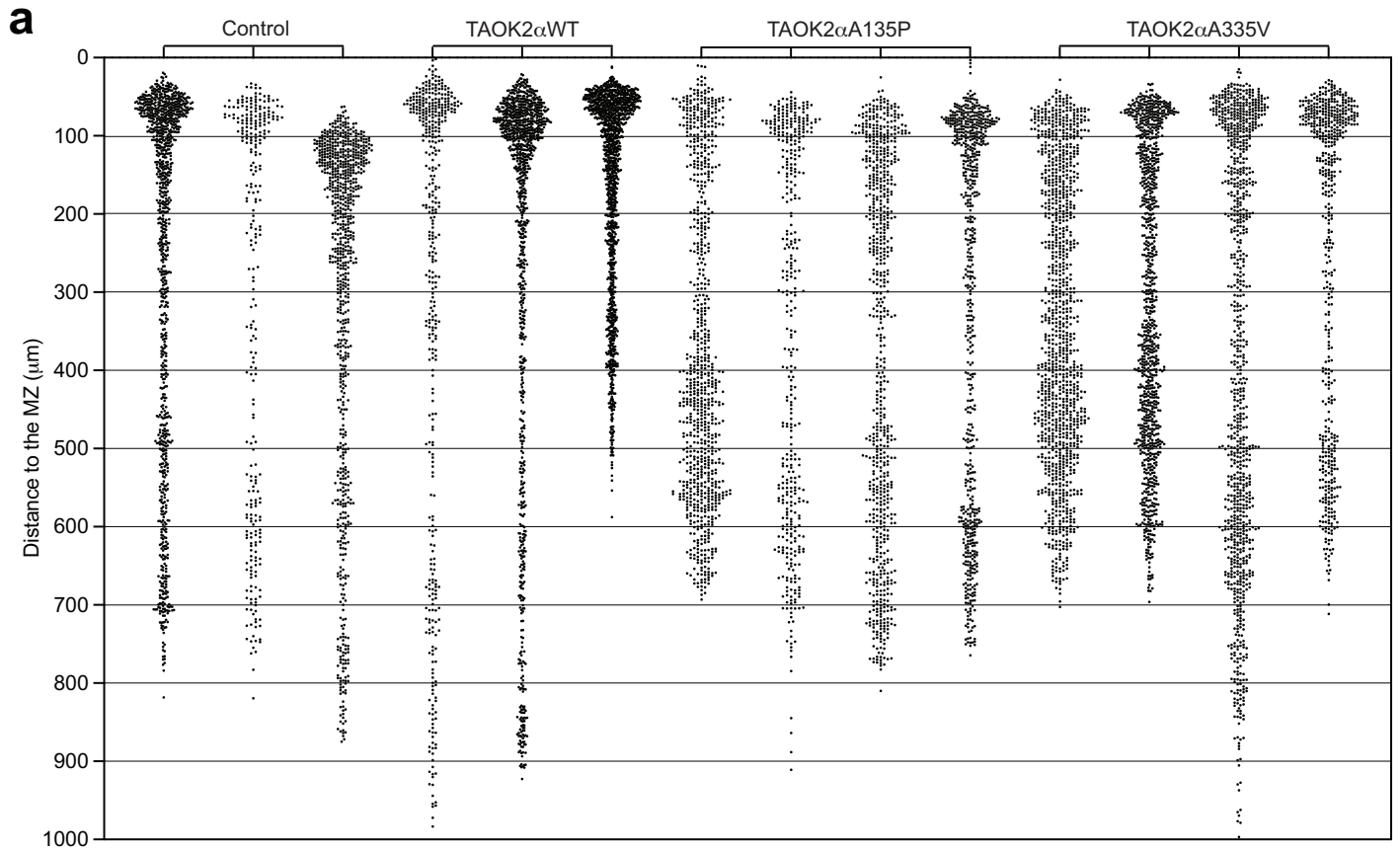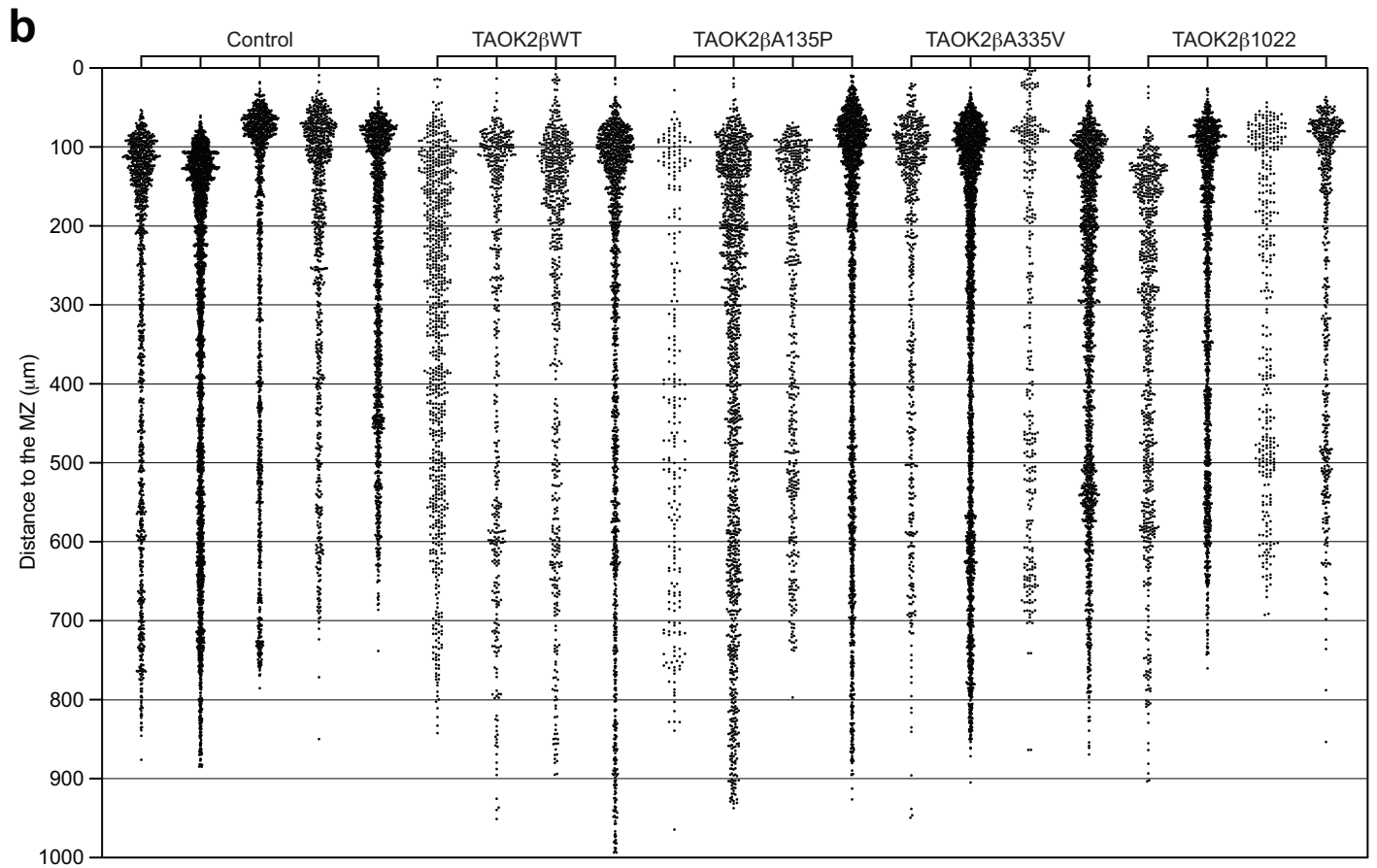

Supplement: Supplementary file 7 — Supp Fig 3 [file 41380_2022_1785_MOESM7_ESM.pdf]

**a**

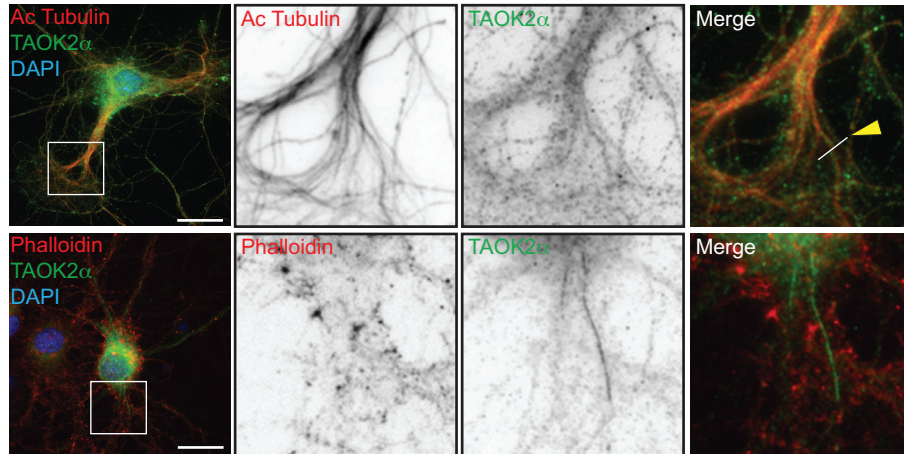

**b**

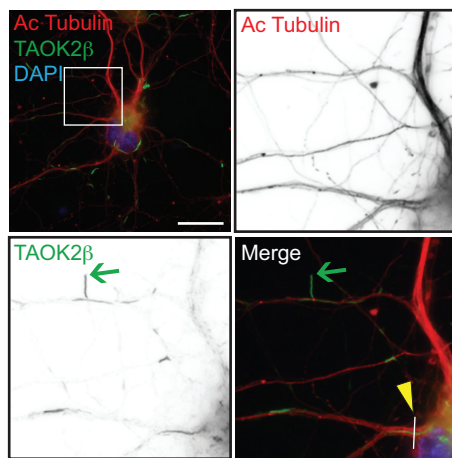

**c**

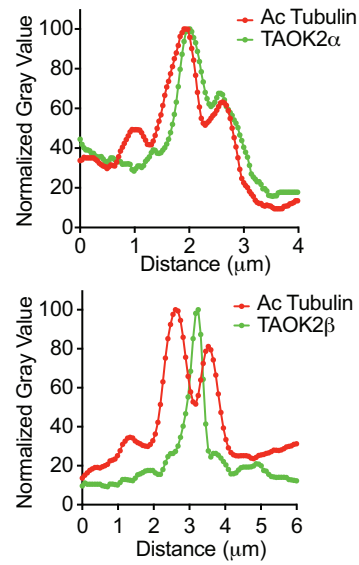

**d**

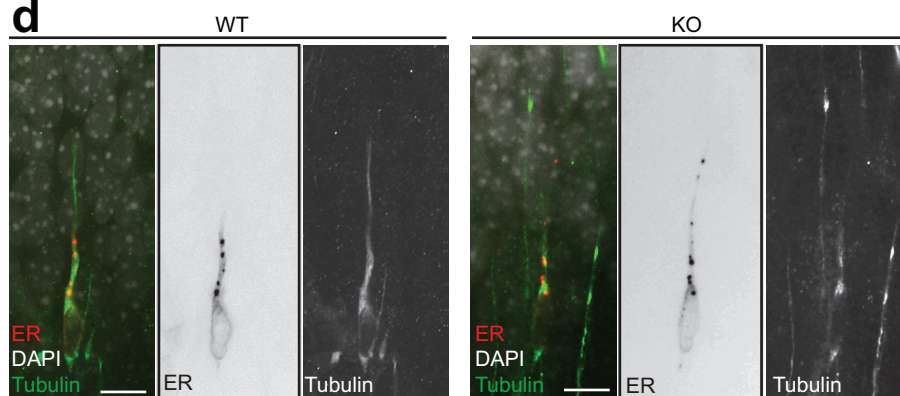

**e**

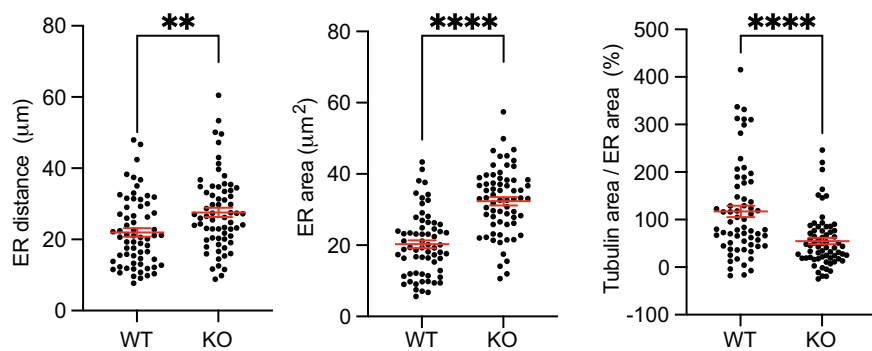

Supplement: Supplementary file 8 — Supp Fig 4 [file 41380_2022_1785_MOESM8_ESM.pdf]

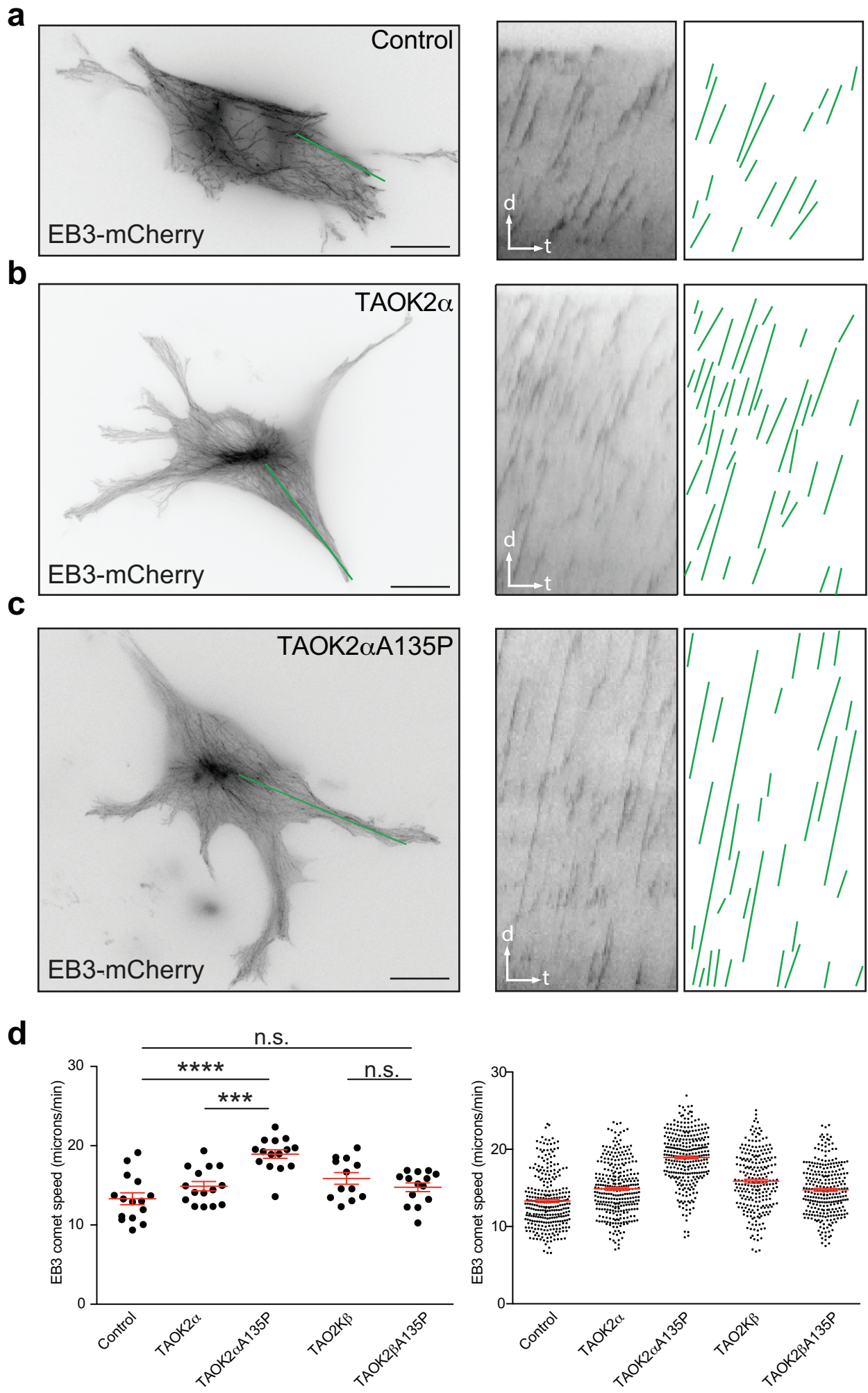

Supplement: Supplementary file 9 — Supp Fig 5 [file 41380_2022_1785_MOESM9_ESM.pdf]

**a**

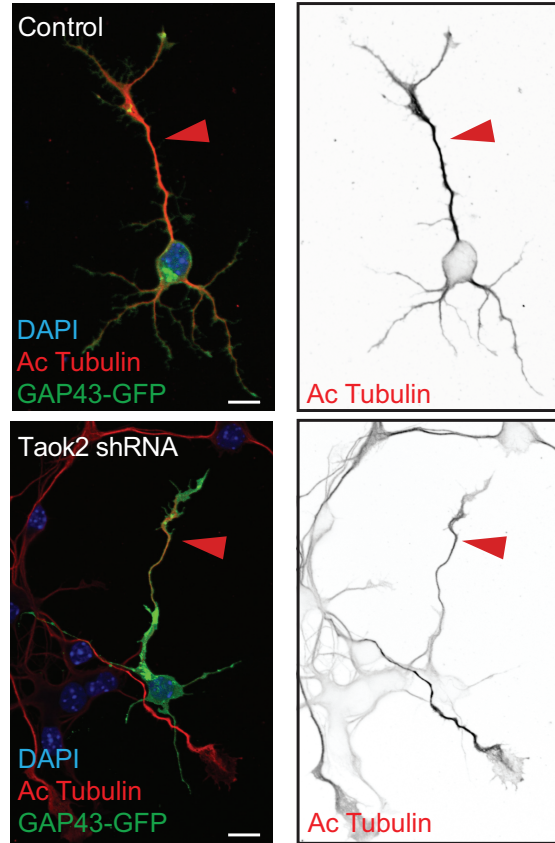

**b**

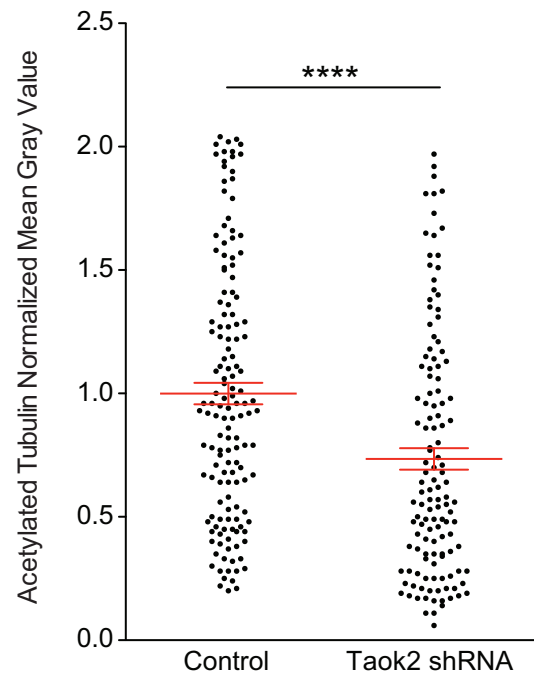

Supplement: Supplementary file 10 — Supp Fig 6 [file 41380_2022_1785_MOESM10_ESM.pdf]

**a**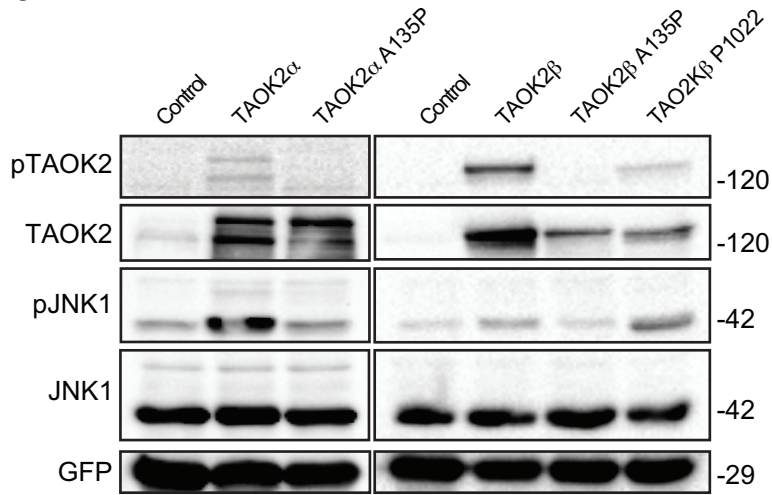**b**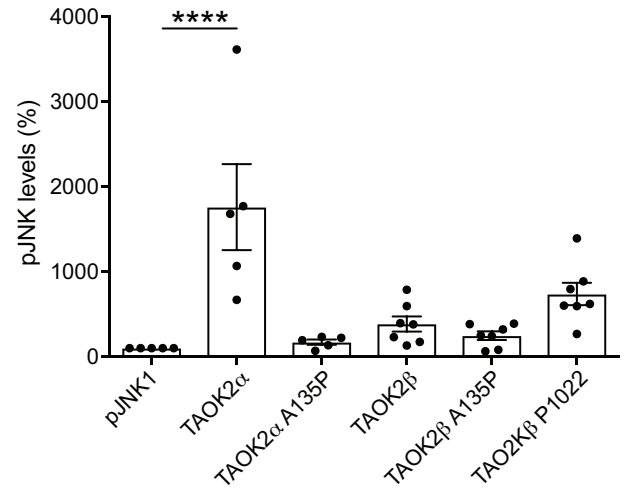

Supplement: Supplementary file 11 — Supp Fig 7 [file 41380_2022_1785_MOESM11_ESM.pdf]

**a**

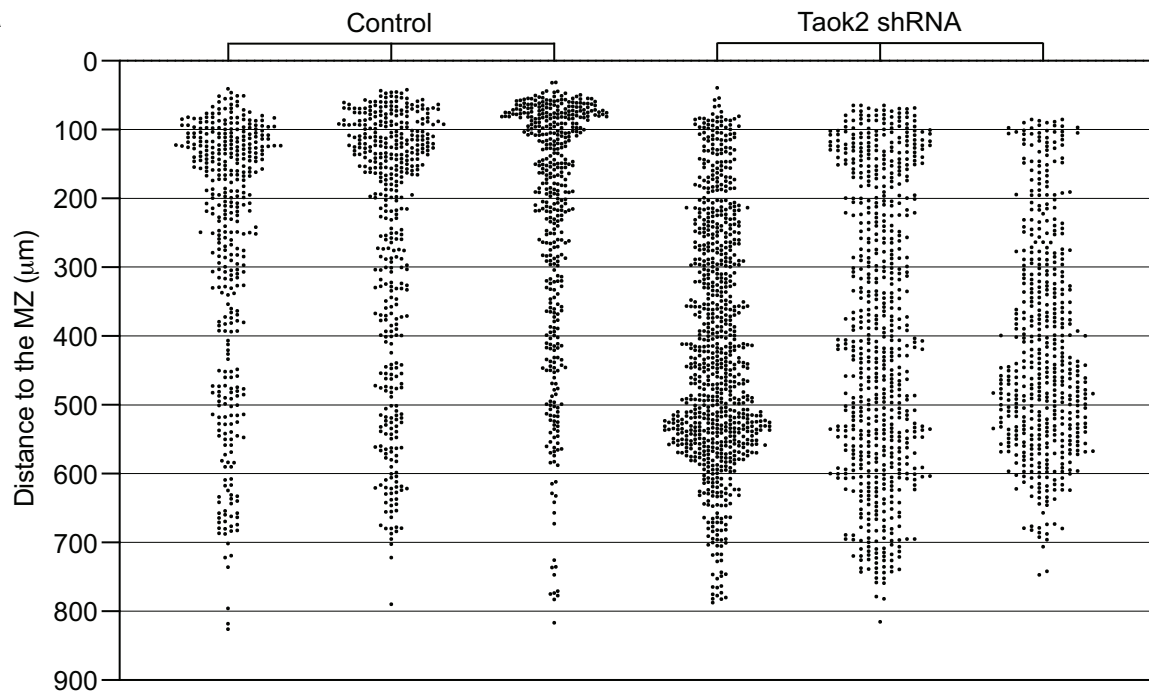

**b**

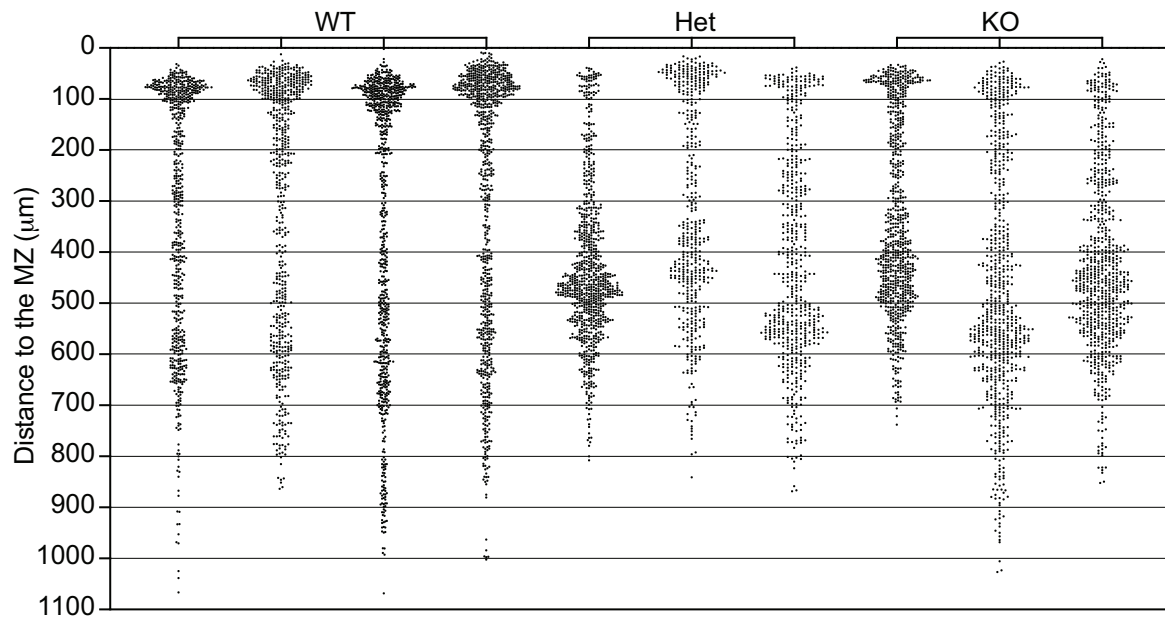

Supplement: Supplementary file 12 — Supp Fig 8 [file 41380_2022_1785_MOESM12_ESM.pdf]

# Supplementary Figure 9

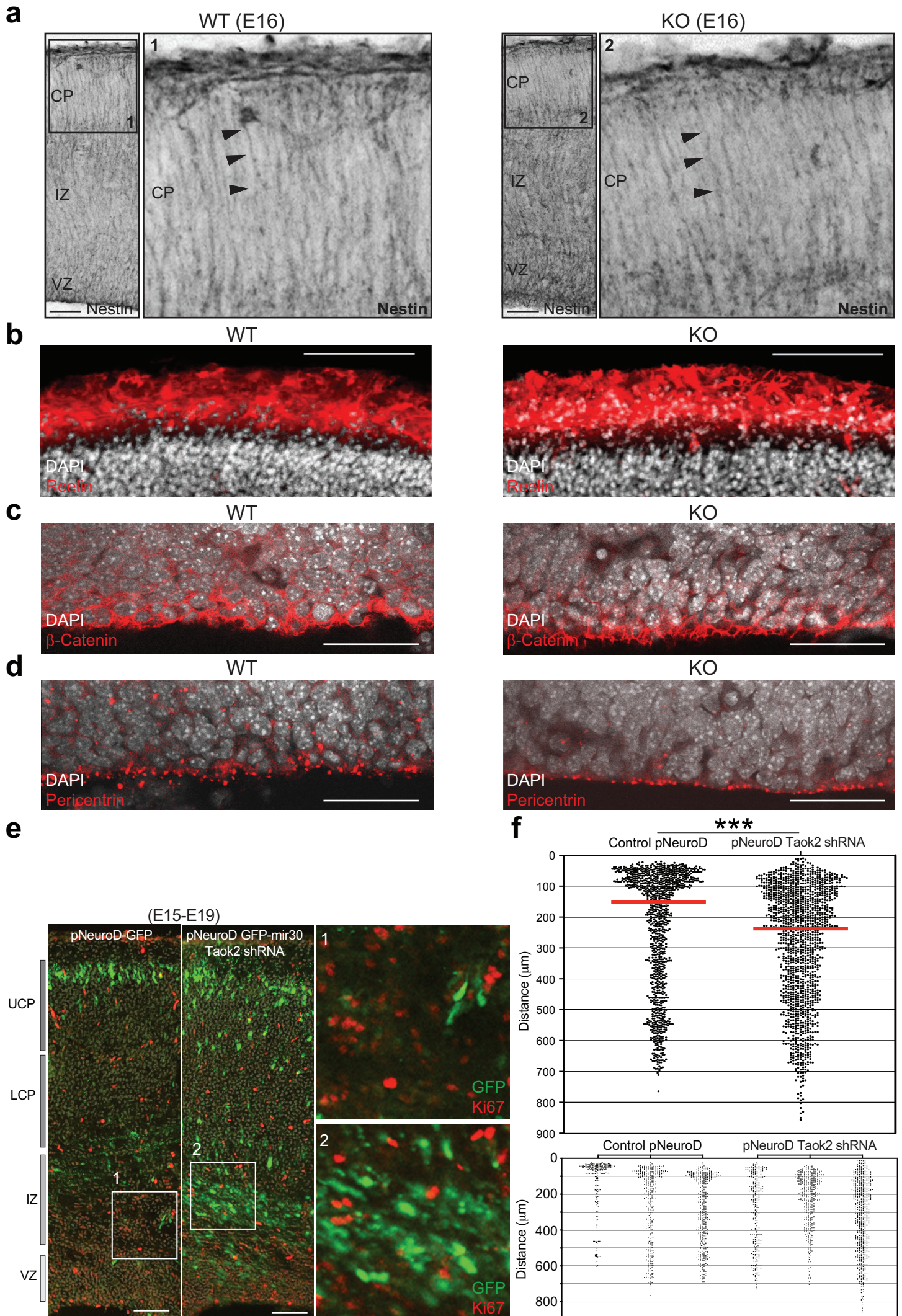

Supplement: Supplementary file 13 — Supp Fig 9 [file 41380_2022_1785_MOESM13_ESM.pdf]

**a**

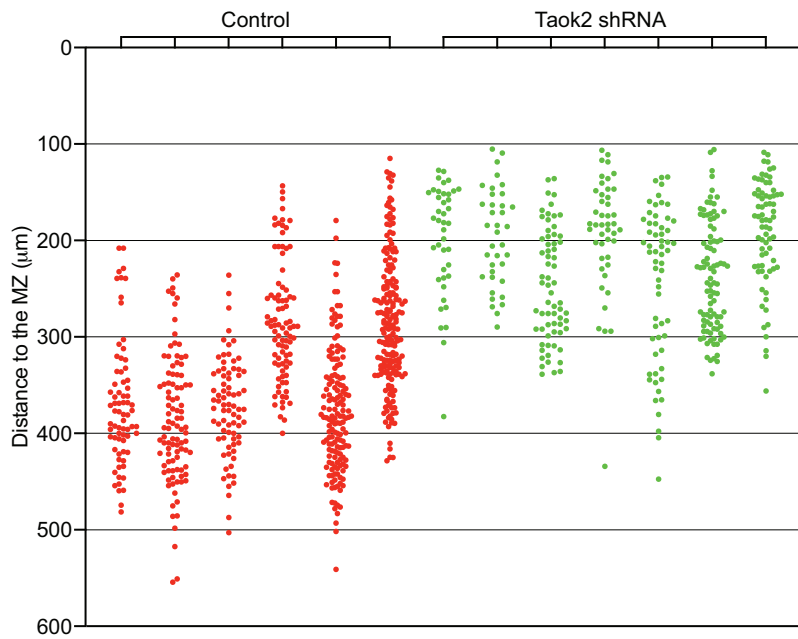

**b**

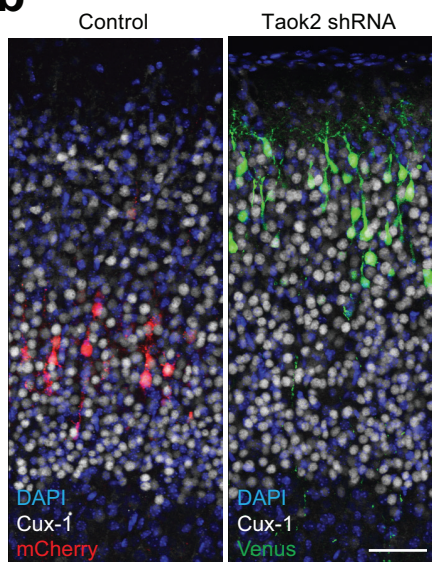

**c**

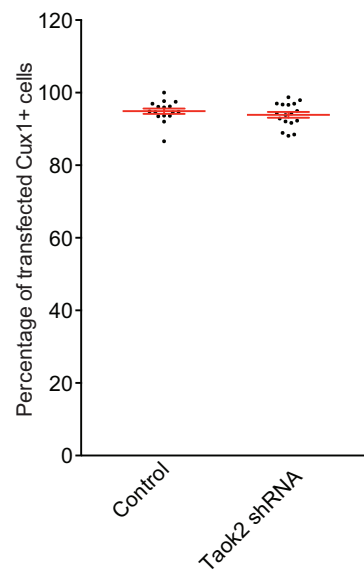

**d**

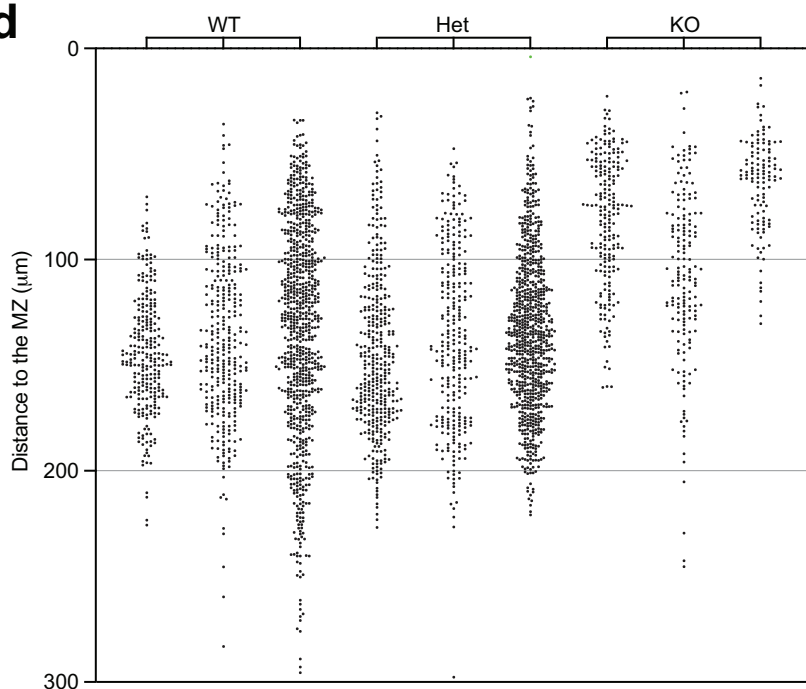

**e**

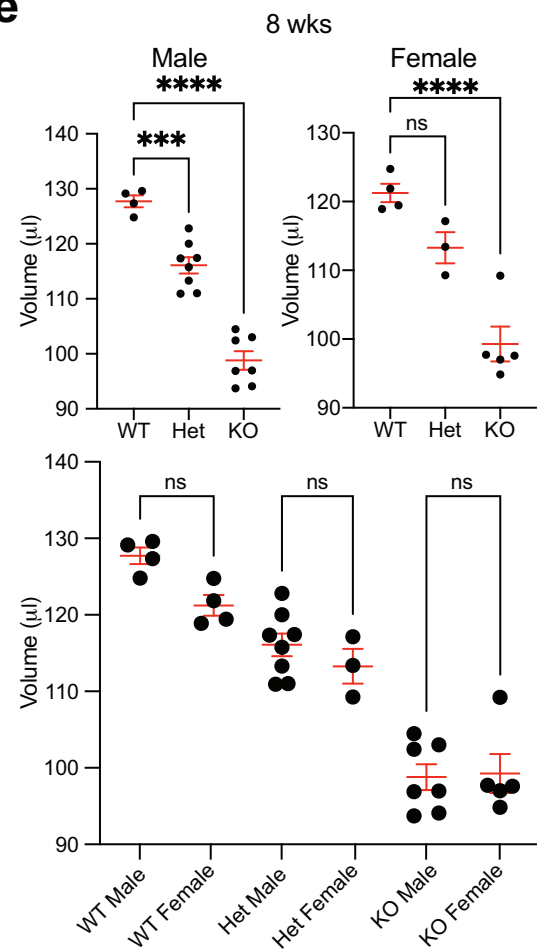

**f**

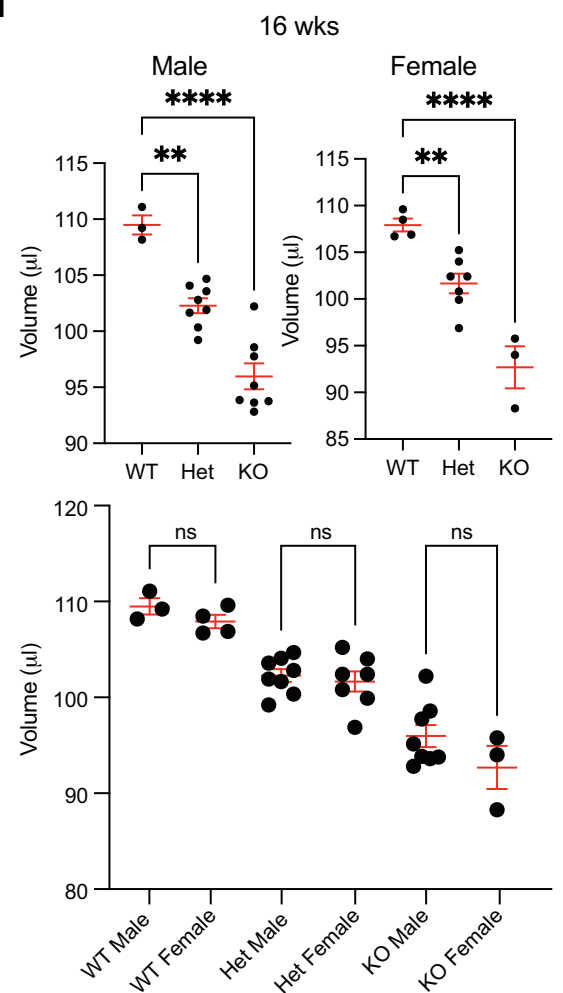

Supplement: Supplementary file 14 — Supp Fig 10 [file 41380_2022_1785_MOESM14_ESM.pdf]

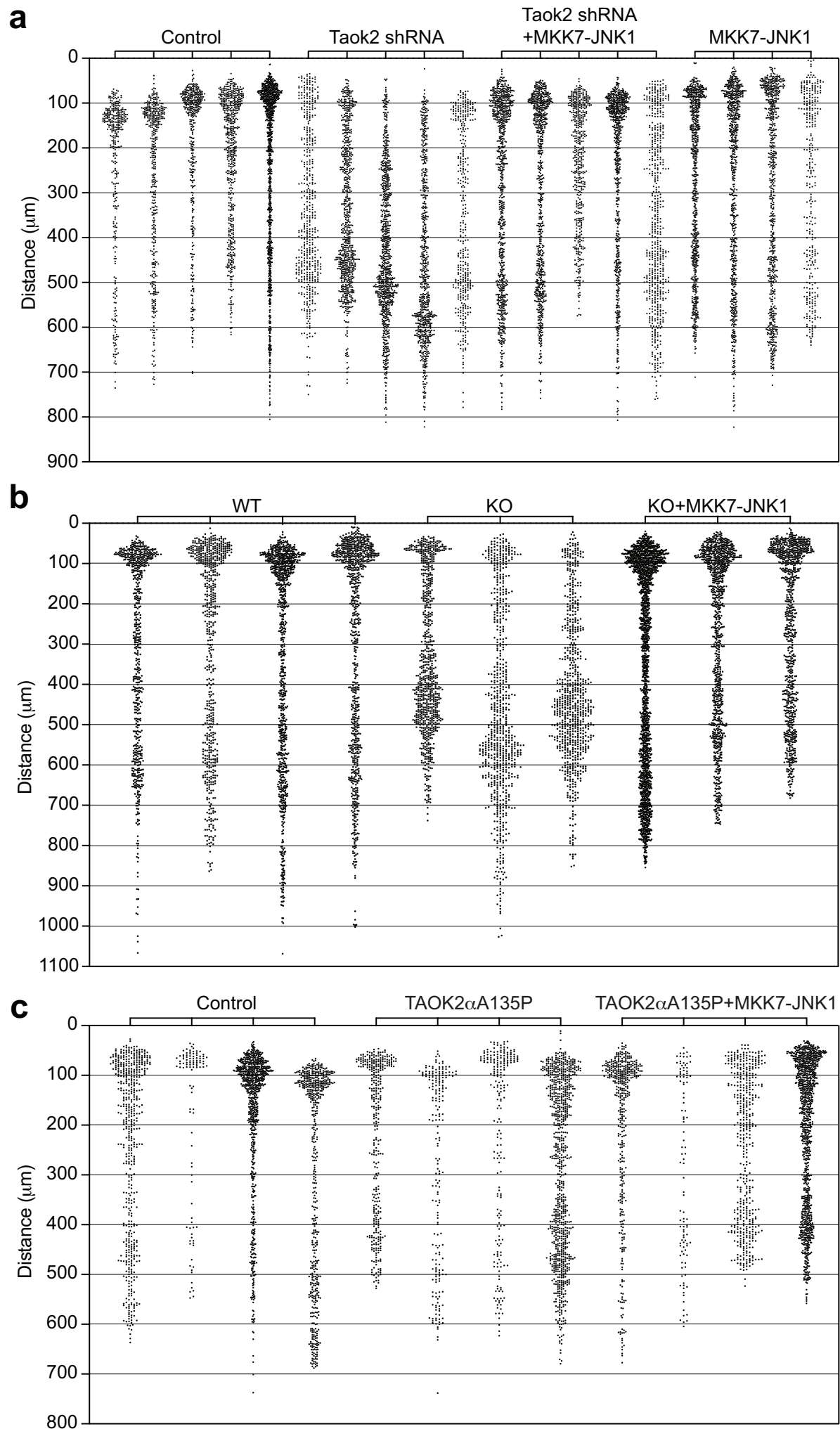

Supplement: Supplementary file 15 — Supp Fig 11 [file 41380_2022_1785_MOESM15_ESM.pdf]

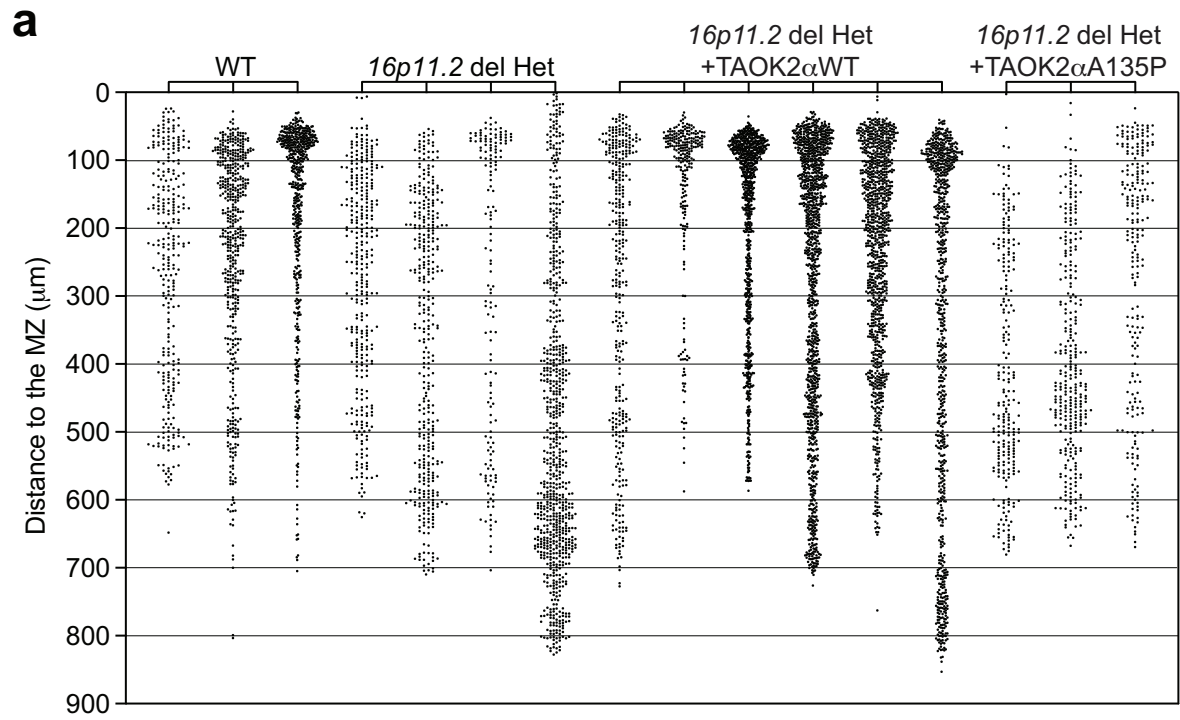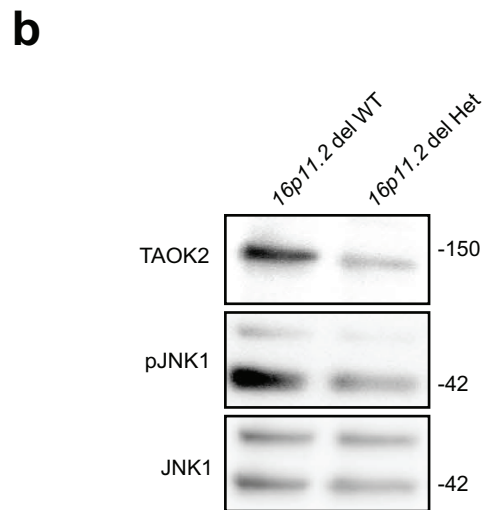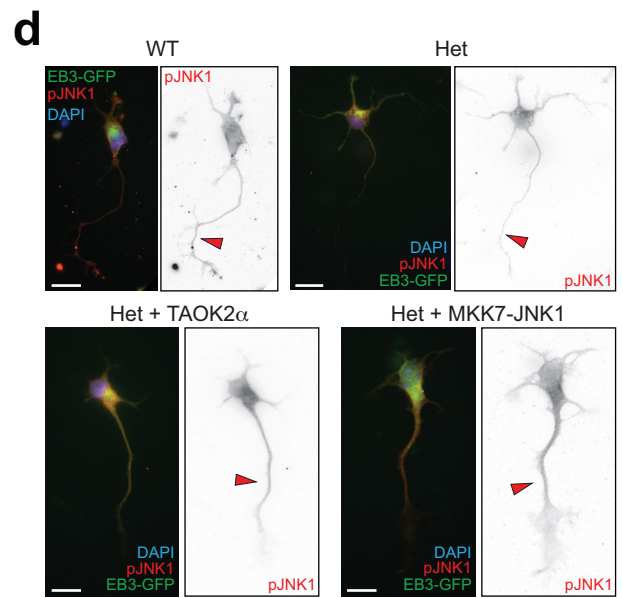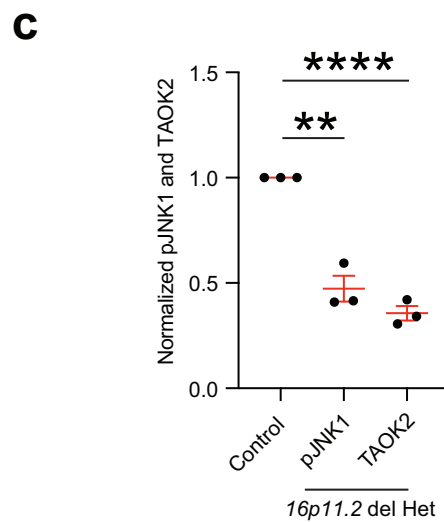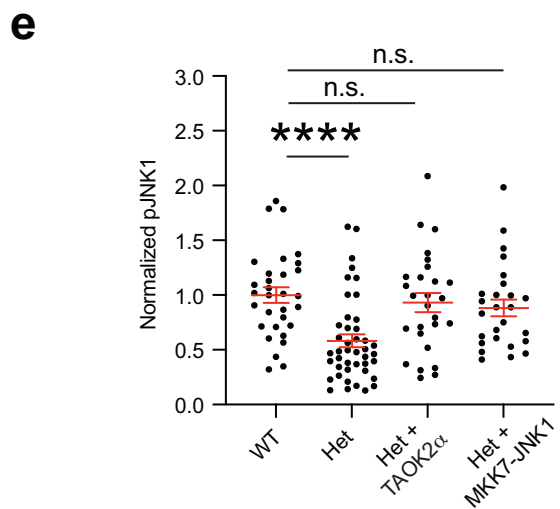

Supplement: Supplementary file 16 — Supp Fig 12 [file 41380_2022_1785_MOESM16_ESM.pdf]
